# Functional Comparison of Chronological and *In Vitro* Aging: Differential Role of the Cytoskeleton and Mitochondria in Mesenchymal Stromal Cells

**DOI:** 10.1371/journal.pone.0052700

**Published:** 2012-12-28

**Authors:** Sven Geißler, Martin Textor, Jirko Kühnisch, Delia Könnig, Oliver Klein, Andrea Ode, Tilman Pfitzner, James Adjaye, Grit Kasper, Georg N. Duda

**Affiliations:** 1 Julius Wolff Institute, Charité – Universitätsmedizin Berlin, Berlin, Germany; 2 Berlin-Brandenburg Center for Regenerative Therapies, Charité – Universitätsmedizin Berlin, Berlin, Germany; 3 Institute for Medical and Human Genetics, Charité – Universitätsmedizin Berlin, Berlin, Germany; 4 Molecular Embryology and Aging Group, Department of Vertebrate Genomics, Max Planck Institute for Molecular Genetics, Berlin, Germany; 5 Institute for Stem Cell Research and Regenerative Medicine, Heinrich Heine University Düsseldorf, Düsseldorf, Germany; Georgia Health Sciences University, United States of America

## Abstract

Mesenchymal stromal cells (MSCs) are of high relevance for the regeneration of mesenchymal tissues such as bone and cartilage. The promising role of MSCs in cell-based therapies and tissue engineering appears to be limited due to a decline of their regenerative potential with increasing donor age, their limited availability in human tissues and the need of *in vitro* expansion prior to treatment. We therefore aimed to determine to which degree *in vitro* aging and chronological aging may be similar processes or if *in vitro* culture-related changes at the cellular and molecular level are at least altered as a function of donor age. For that purpose we established MSCs cultures from young (yMSCs) and aged (aMSCs) rats that were cultured for more than 100 passages. These long-term MSCs cultures were non-tumorigenic and exhibited similar surface marker patterns as primary MSCs of passage 2. During *in vitro* expansion, but not during chronological aging, MSCs progressively lose their progenitor characteristics, e.g., complete loss of osteogenic differentiation potential, diminished adipogenic differentiation, altered cell morphology and increased susceptibility towards senescence. Transcriptome analysis revealed that long-term *in vitro* MSCs cultivation leads to down-regulation of genes involved in cell differentiation, focal adhesion organization, cytoskeleton turnover and mitochondria function. Accordingly, functional analysis demonstrated altered mitochondrial morphology, decreased antioxidant capacities and elevated ROS levels in long-term cultivated yMSCs as well as aMSCs. Notably, only the MSC migration potential and their antioxidative capacity were altered by *in vitro* as well as chronological aging. Based on specific differences observed between the impact of chronological and *in vitro* MSC aging we conclude that both are distinct processes.

## Introduction

Mesenchymal stromal cells (MSCs) are highly proliferative cells that are able to home to and engraft in different tissues and finally differentiate into functional osteoblasts, chondrocytes and/or adipocytes [1]. Their healing-promoting properties result not only from their ability to differentiate into functional mesenchymal cells, but also from their paracrine effects. For instance MSCs serve as source of cytokines and proteinases essential to angiogenesis and matrix-remodeling such as VEGF, MMPs, TGF-β, and bFGF [2], [3]. Advantageously, MSCs can be directly obtained from patient's bone marrow or adipose tissue, thereby avoiding ethical and safety issues associated with the use of embryonic stem cells (ESCs) or induced pluripotent cells (iPSC). Thus, MSCs are thought to be an attractive cell source for cell-based therapies and tissue engineering. In experimental approaches the regenerative capability of MSCs has been validated for femoral head necrosis, osteogenesis imperfecta, large bone defects, infantile hypophosphatasia, GVHD, cartilage defects and tendon repair [4]–[7].

Even though MSCs therapies have been successful *in vitro* and in animal settings, a broad clinical application of such therapies is still missing [1]. One reason may be that in mammals the regeneration potential of mesenchymal tissues declines with age, which might be at least partially due to age-related changes in MSC quantity and quality [8], [9]. We and other groups demonstrated that chronological aging of the donor is associated with a decline of MSC number, reduced migration potential and diminished differentiation capacity [10], [11]. On the molecular level these changes in cellular function were attributed to decreased cytoskeleton turnover, lower antioxidant activity and higher susceptibility towards senescence.

Similarly, also extended MSC expansion *in vitro* seems to compromise their regenerative function. In this regard, earlier studies already questioned the capability of endless MSC expansion, which may result in loss of progenitor properties and in malignant transformation [12], [13]. This indicates that MSC-based therapeutic strategies require reliable markers for phenotypic, functional and genetic characterization of employed cell population after *in vitro* expansion.

Since both individual chronological (*in vivo*) aging and *in vitro* aging, due to long-term cultivation, affect MSCs characteristic, the question arises to which degree these two processes differ and in which respect they may be similar. Recently, it has been hypothesized that chronological and *in vitro* aging of human MSCs induce similar alterations in gene expression [14]. Thus, the aims of this study are to determine a) to which extent *in vitro* and *in vivo* aging are related processes leading to similar cellular and molecular alterations, and b) if long-term culture-related changes are altered as a function of the chronological age.

## Materials and Methods

### Ethics Statement

All experiments involving the use of animals were in compliance with the German Animal Welfare Act (TierSchG §4 [3]) and were approved by State Office of Health and Social Affairs Berlin (Permit Number: IC113-Reg 0232/07).

### MSC isolation

MSCs were isolated from the bone marrow of three week and 12 months old male Sprague-Dawley rats (Harlan Winkelmann, Germany, www.harlan.com), selected by plastic adherence and cultured in expansion medium (EM) [10]. Culture medium was substituted twice a week and cells were harvested after reaching 70–80% confluence using trypsin. Cell number and cell diameter distribution of trypsinized MSCs were determined using the cell counter CASY TT (Roche, Germany, www.roche-applied-science.com). The MSC cell surface marker expression was validated using flow cytometry with specific antibodies (Table S1) as previously described [15].

### Functional assays

#### Proliferation

For short term proliferation assays, 2000 MSCs/cm^2^ were seeded onto 96-well plates (96-MTP). Cell number was measured one and four days after seeding using CyQuant® assay (Promega, Germany, www.promega.com) according to manufactures instruction.

#### Migration

Modified Boyden chamber assay was performed as described elsewhere [10]. Briefly, 1×10^4^ MSCs were seeded and incubated for 5 h at 37°C. Non-migrated cells were removed from the upper side; remaining cells stained with 10 µg/ml Hoechst-33342 (Invitrogen, Germany, www.invitrogen.com) and counted in ten microscope regions per filter (10× magnification), for two filters per sample. The average numbers of migrated cells were analyzed using the NIH ImageJ (http://rsb.info.nih.gov/nih-image/).

#### Differentiation

Osteogenic differentiation of confluent MSCs was induced by using osteogenic media (OM) [10] supplemented either with dexamethasone or BMP2. The matrix mineralization was visualized with Alizarin Red staining. Quantification was achieved by measuring the absorbance of Alizarin Red (OD_AR_) that was normalized to cell number determined by alamarBlue® (OD_AB_) (Invitrogen). Adipogenic differentiation was induced by using adipogenic medium (AM) [10] and quantified after Oil red O (OR) staining which was normalized to cell number.

### Western blot

The Novex® system was employed according to the Invitrogen NuPAGE® protocol. Primary antibodies were mouse(α-rat CDKN2A/p16^INK4a^), mouse(α-rat CDKN1A/p21^WAF1/Cip1^) (1∶1000, Abcam, Germany, www.abcam.com) and mouse(α-rat GAPDH) (1∶7000, Abcam). As secondary antibody goat(α-mouse IgG)peroxidase was utilized. Band intensities were quantified by NIH ImageJ software package (http://rsb.info.nih.gov/ij/).

### Anchorage-independent growth assay

Soft agar assay was used in order to investigate cell transformation of long-term cultivated MSCs. The assay was performed as previously described [16]. Briefly, the assay consists of a lower layer (1.2% agar) and an upper layer (0.6% agar) in a 6-well plate. The cells were suspended in the upper layer. The assay was incubated at 37°C and 5% CO2 for 2–3 weeks. Subsequent, the plates was stained with 0.2% neutral red for 1 h. After washing with PBS, colonies were counted directly using a microscope.

### llumina Bead Chip Hybridization

Total RNA was isolated using Trizol® (Invitrogen) reagent as describes previously [17] and purified using Qiagen RNeasy® mini kit (Qiagen, Germany, www.qiagen.com) according to manufacturers instruction. Illumina® BeadChip hybridization was performed as described elsewhere [18]. Briefly, biotinylated cRNA was produced from 500 ng total RNA using Illumina® TotalPrep™ RNA amplification kit (Invitrogen). Illumina® RatRef-12 Expression BeadChips hybridization, washing, Cy3 streptavidin staining, and scanning were performed using Illumina® BeadStation 500 platform. Basic expression data analysis was carried out using the BeadStudio software 3.0. Raw data were background-subtracted and normalized using the “rank invariant” algorithm and then filtered for significant expression on the basis of negative control beads. Significant detection of a gene within a sample group was assessed at a detection p≤0.01. Significant regulation of a gene between two groups was assumed at differential p<0.05 and an expression ratio ≥1.5. Functional categorizing of all differentially expressed mRNAs was performed using the Database for Annotation, Visualization and Integrated Discovery (david.abcc.ncifcrf.gov) [19].

The microarray data is available at Gene Expression Omnibus (http://www.ncbi.nlm.nih.gov/geo/query/acc.cgi?token=nvmlfscciuoocbk&acc=GSE36596) under the accession number GSE36596.

### Quantitative real-time polymerase chain reaction (qRT-PCR)

Isolated RNA was reverse transcribed using the iScript™ cDNA Synthesis Kit (BioRad, Germany, www.biorad.com) according to manufacturer's instructions. The qRT-PCR was performed in the iQ™5 Real-Time PCR Detection System (BioRad) using iQ™ SYBR® Green Supermix (BioRad) as described in [17]. All primers employed were intron spanning and their sequences are provided in Table S2. Quantification of mRNA expression of each gene was calculated with the comparative Cycle Threshold (Ct) method normalized with the housekeeping gene.

### Immunocytochemistry

Mitochondria network were stained using MitoTracker® Red CM-H_2_XRos (MTR) (Invitrogen). Cells were plated into chamber slides one day prior the staining. Cells were incubated with 300 nM MTR for 30 min at 37°C. Subsequently the cells were fixed using 4% paraformaldehyd and permeabilized with 0.1% saponin dissolved in PBS. Visualization of actin fibers and mitochondria of fixed and permeabilised cells was achieved by incubation with Alexa 594-conjugated phalloidin (6.6 nM; Invitrogen) or with specific mouse(α-rat Cytochrome C) antibody (BD Biosciences, Germany, www.bdbiosciences.com). Nuclei were stained with DAPI and goat(α-mouse IgG)-488 (Invitrogen) was used as secondary antibody. Fluorescence imaging was performed with a Leica DMI6000B live cell microscope system (Leica, Germany, www.leica.com) under identical excitation and exposure conditions. Cell area and cell roundness as well as mitochondria network area were quantified using Columbus 2.0 software (PerkinElmer, Germany, www.perkinelmer.de) and results are presented as mean ± standard error of the mean (SEM). Each experiment was conducted in triplicates and approximately 200 cells/sample were measured.

### Reactive oxygen species (ROS) measurement

Concentrations of ROS were determined using CM-H2-DCFDA (Invitrogen). Two days prior the experiment, 2000 cells were seeded per well of a 96-MTP and cultivated in EM. Subsequently, cells were washed twice with PBS and incubated with EM supplemented with 10 µM CM-H2-DCFDA for 20 min at 37°C. The resulting fluorescent signal was measured using Infinite® 200Pro plate reader (Tecan, Germany, www.tecan.com). Cells pretreated with 200 nM pyocyanin for 20 min were used as positive control. The fluorescent intensities were normalized to the cell number determined by CyQuant®.

### ATP-measurement

Cellular ATP was determined using ATPLite™ bioluminescence luciferase-based assay (Perkin Elmer) as previously described [18]. Briefly, 2000 MSCs/cm^2^ were seeded per well of a 96-MTP and cultured for 3 days. Subsequently, assay was performed according to manufacturer's instructions. Luminescence values were quantified using the provided ATP standard solution. Obtained values were normalized to cell number determined by CyQuant®.

### Antioxidant activity assay

Total antioxidant activity of cell lysates was investigated by the Trolox® equivalent antioxidant assay kit (Sigma-Aldrich, Germany, www.sigmaaldrich.com) according to manufacturer's instructions. Briefly, cells were seeded in 6-well plates and cultivated for three days. Subsequently, cell lysates were generated as described elsewhere [10] and total protein concentrations were measured. Each cell lysate were measured in triplicates using 20 µg total protein. Absorbance values were quantified against a Trolox® standard row.

### Mitochondrial membrane potential (Δψm)

Alterations in the ΔΨm were measured using MitoProbe™-JC-1 (Invitrogen, Germany) according to manufacturer's instruction. Briefly, cells were seeded on a 96-MTP and cultured for one day in EM. Subsequently, cells were incubated with 20 µM MitoProbe™-JC-1 for 30 min at 37°C in the dark. Medium was removed and cells were washed twice with PBS. The ratio of red to green fluorescence from JC-1 was quantified using Infinite® 200Pro. MSCs pre-treated with 25 µM Valinomycin served as controls for dissipation of ΔΨm.

### Statistical analysis

The SPSS 18.0 software package (SPSS Inc., Chicago, IL, USA) was used for statistical evaluation. If not stated otherwise, results from at least four independent experiments were analyzed for statistical significance using the Student's t-test. Multiple pairwise comparisons were performed by one-way analysis of variance (ANOVA, repeated measures) and p-values were adjusted using Bonferroni's p-value adjustment multiple comparison procedure. Unless otherwise specified, results are presented as mean ± standard deviation (SD). All tests were analyzed two-sided and p<0.05 was regarded as significant.

## Results

### Generation of *in vitro* aged MSCs

**Figure 1:**
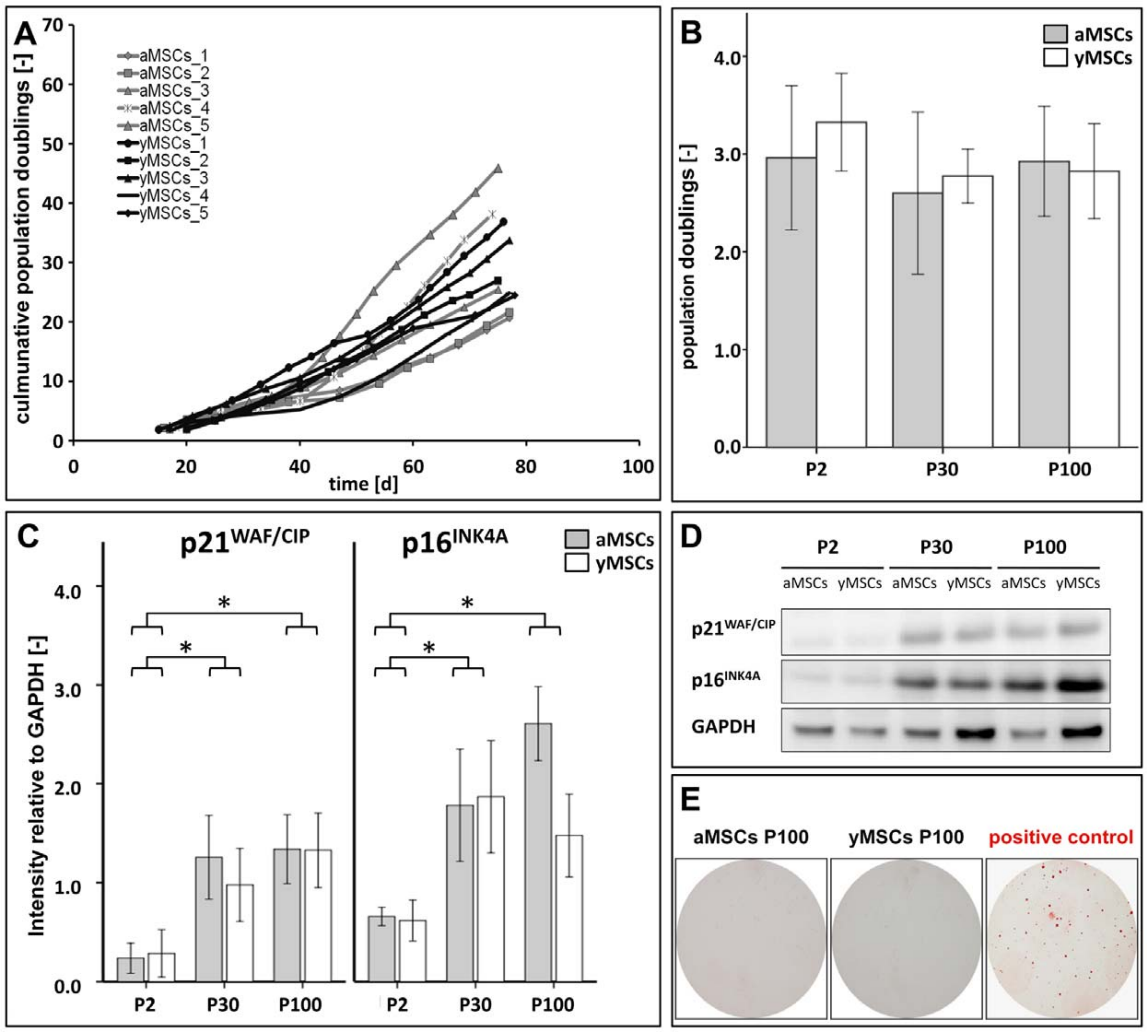
Generation and characterization of *in vitro* aged MSCs. (**A**): Cumulative population doublings of aMSCs and yMSCs during the first 80 days of culture are shown (n = 5). (**B**): Long-term cultivation has no influence on short-term proliferation rate of aMSCs and yMSCs of passage 30 and 100. Proliferation assay was performed using CyQuant®. (**C**): Graphs illustrate quantified signal intensities of p21^WAF1/CIP1^ and p16^INK4A^ relative to GAPDH. (**D**): Representative Western blots showing increased p21^WAF1/CIP1^ and p16^INK4A^ expression during *in vitro* aging. GAPDH served as endogenous control. (**E**): In anchorage-independent growth assays *in vitro* aged MSCs_P100_ did not form colonies, while the breast carcinoma cell line MDA-MB-231, which served as positive control, produced numerous colonies (n = 3). Abbreviations: aMSCs, mesenchymal stromal cells from aged donors; yMSCs, mesenchymal stromal cells from young donors; P: passage. * indicates statistical significance (p<0.05).

In order to establish *in vitro* aged MSC cultures from young (3 weeks) and aged (12 months) SD-rats, isolated MSCs were sub-cultured under standard cell culture conditions until passage 100 (P100). Unexpectedly, long-term culture of MSCs from aged (aMSCs) and young (yMSCs) rats did not diminish their proliferation rate as indicated by the number of population doublings (PD) per passage (Figure 1A). Both, aMSCs and yMSCs exhibited a similar proliferation rate throughout the long-term culture (±SD: PD_aMSC_ = 2.7±0.8; PD_yMSCs_ = 2.4±0.3; p = 0.450). All MSC cultures were maintained for more than 100 passages without ultimately reaching the state of cell cycle arrest.

To further characterize the background of the *in vitro* aging process, a pair of long-term cultivated aMSCs and yMSCs of P100 (aMSCs_P100_; yMSCs_P100_) and P30 (aMSCs_P30_; yMSCs_P30_) were functionally and biochemically investigated and compared to primary MSCs of P2 (aMSCs_P2_; yMSCs_P2_). No differences in proliferation between primary and *in vitro* aged aMSCs and yMSCs of P2, P30, and P100 were determined (Figure 1B). However, Western Blot analysis revealed a significant increased expression of the cell cycle inhibitors p21 and p16 in long-term cultivated aMSCs and yMSCs of P30 and P100 compared to aMSCs and yMSCs of P2 (Figure 1C and D).

The tumorigenic potential of *in vitro* aged MSCs was estimated by an anchorage-independent growth assay. In contrast to the breast carcinoma cell line MDA-MB-231, which served as positive control, *in vitro* aged MSCs_P100_ showed no growth in soft agar (Figure 1E) indicating a non-transformed status.

**Figure 2:**
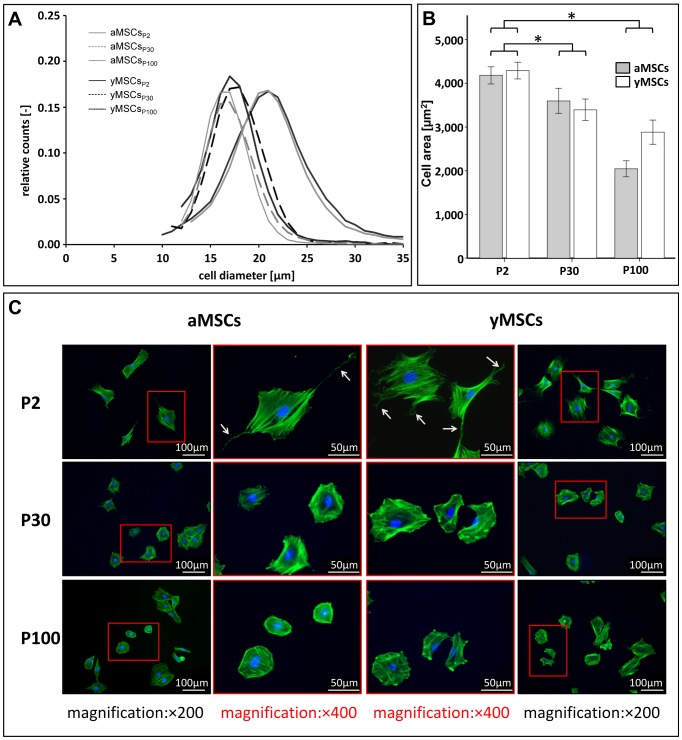
Long-term *in vitro* culture alters MSC morphology independent from the donor age. (**A**): Cell diameter of aMSCs and yMSCs decreases during the course of long-term cultivation. Diagram shows the cell size distribution of MSCs measured by CASY® TT cell analyzer system at indicated passages after trypsinization. (**B**): Cellular area of attached aMSCs and yMSCs significantly decreases during *in vitro* aging. Measurements were performed from fluorescence images of identical exposure conditions. (**C**): Representative images of phalloidin labeled MSCs highlight reduction of cellular expansion. Additionally, *in vitro* aged aMSCs and yMSCs exhibited less filopodia, lamellipodia and cell spreading (white arrows). * indicates statistical significance (p<0.05).

Next, we analyzed alterations in morphology of MSCs upon *in vitro* aging. During the course of long-term cultivation the cell diameter of aMSCs and yMSCs decreases (aMSCs: mean_P2_ = 19±5 µm; mean_P30_ = 16±3 µm, p_P2 vs. P30_<0.001; mean_P100_ = 16±3 µm, p_P2 vs. P100_<0.001; yMSCs: mean_P2_ = 20±5 µm; mean_P30_ = 17±3 µm, p_P2 vs. P30_<0.001; mean_P100_ = 16±3 µm, p_P2 vs. P100_<0.001; Figure 2A). Morphological analysis of fixed and phalloidin stained MSCs demonstrated a significantly larger cell area of aMSCs_P2_ (mean_P2_ = 4182±100 µm^2^) and yMSCs_P2_ (mean_P2_ = 4291±97 µm^2^) compared to their *in vitro* aged counterparts (aMSCs: mean_P30_ = 3597±144 µm^2^, p = 0.019; mean_P100_ = 2045±92 µm^2^, p = 0.001; yMSCs: mean_P30_ = 3394±124 µm^2^, p<0.001; mean_P100_ = 2881±140 µm^2^, p<0.001) (Figure 2B). The observed morphological changes were not only restricted to the cell size. Compared to the primary MSCs_P2_, *in vitro* aged aMSCs and yMSCs exhibited also less filopodia and lamellipodia, diminished cell spreading and increased cellular roundness (Figure 2C, Figure S1).

### Long-term cultivation adversely affects differentiation and migration potential of MSCs

In order to analyze the influence of *in vitro* aging on the MSCs phenotype, their cell surface marker patterns were determined by flow cytometry. In line with recent findings MSC cell surface markers CD29, CD44, CD73, CD90, CD105, CD106, CD166, and RT1A were expressed on *in vitro* aged aMSCs_P100_ and yMSCs_P100_ (Figure S2). Moreover, *in vitro* aged aMSCs_P100_ and yMSCsP_100_ were negative for CD45, CD34 and RT1B.

**Figure 3:**
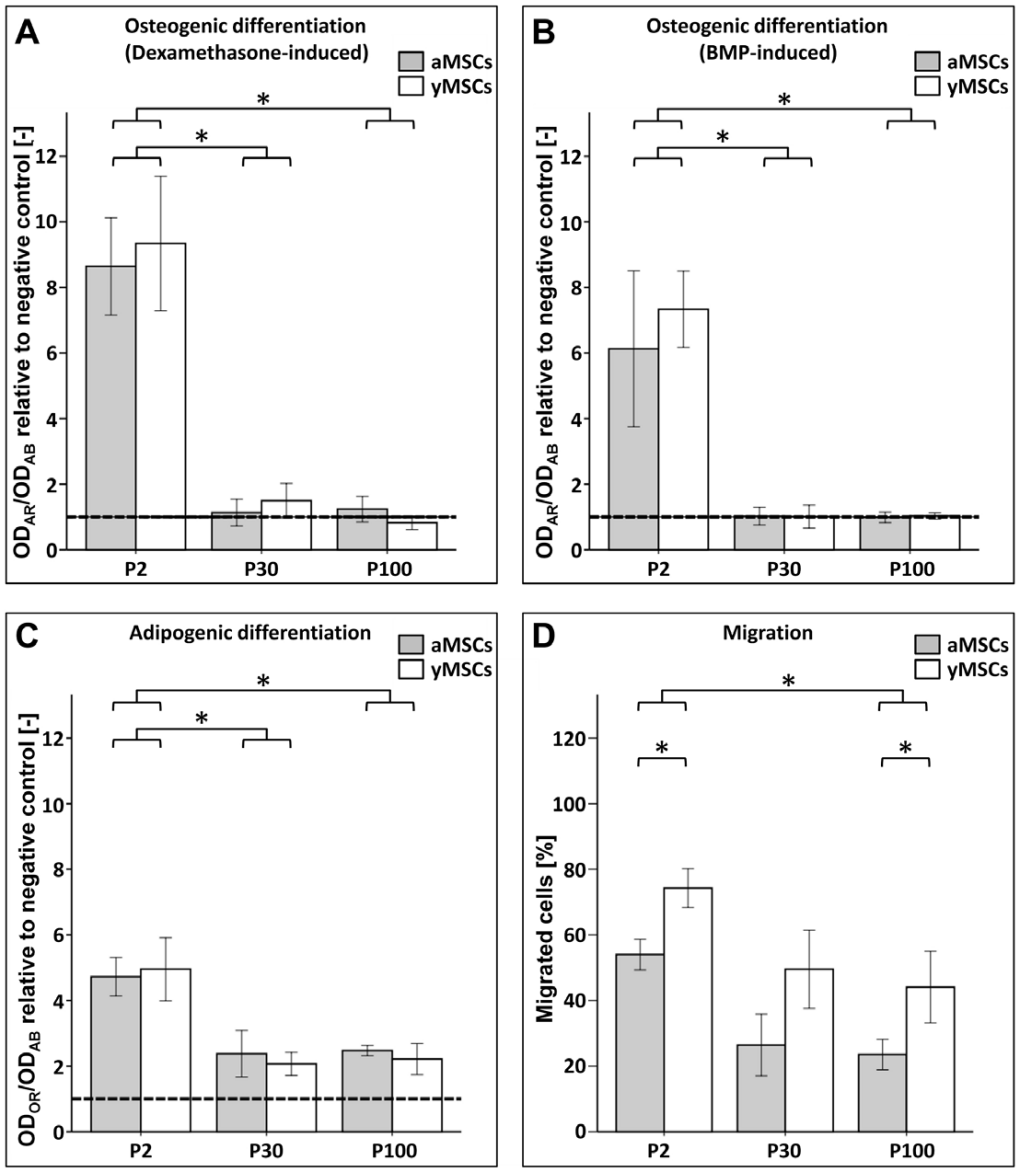
Long-term cultivation negatively influences the differentiation and migration potential of aMSCs and yMSCs. (**A**): In contrast to primary MSCs of passage 2, *in vitro* aged aMSCs and yMSCs of P30 and P100 show no matrix mineralization. Osteogenic differentiation was initiated with dexamethason and determined by matrix mineralization (Alizarin Red, AR) and normalized to cell number (alamarBlue®, AB). Dashed lines indicate differentiation potential of the negative control cultured in EM. (**B**): Under stimulation with BMP2, aMSCs and yMSCs of P2 show strong osteogenic differentiation, while again no matrix mineralization was observed in long-term MSC cultures of P30 and P100. (**C**): Adipogenic differentiation of aMSCs and yMSCs of P30 and P100, induced by adipogenic medium, was diminished by 50% compared to aMSCs and yMSCs of P2. In reference to the negative control maintained in EM (dashed line), aMSCs and yMSCs of P30 and P100 retained a potential for adipogenic differentiation. Differentiation was determined by using Oil red O (OR) staining and normalized to cell number. Diagram shows values normalized to negative control. (**D**): The number of migrated cells declined with increased *in vitro* passage. Moreover, aMSCs of each passage demonstrated significantly lower migratory potential compared to yMSCs. Migration rates were measured with a modified Boyden chamber assay. At least five independent experiments were carried out for all assays. Abbreviations: OD, optical density. * indicates statistical significance (p<0.05).

The MSC differentiation potential was tested by stimulation with osteogenic (OM) and adipogenic media (AM). Cells cultured in expansion medium (EM) served as negative control. In contrast to MSCs_P2_, *in vitro* aged MSCs_P30_ and MSCs_P100_ cultured in OM supplemented with dexamethasone showed no matrix mineralization (OD_AR_/OD_AB_ relative to negative control: mean_aMSCsP2_ = 8.6±1.5; mean_aMSCsP30_ = 1.1±0.4, p_P2 vs. P30_<0.001; mean_aMSCsP100_ = 1.24±0.4, p_P2 vs. P100_<0.001; mean_yMSCsP2_ = 9.3±2.1; mean_yMSCsP30_ = 1.5±0.5, p_P2 vs. P30_<0.001; mean_yMSCsP100_ = 0.82±0.21, p_P2 vs. P100_<0.001) (Figure 3A). This loss in osteogenic differentiation capacity appeared to be independent from donor age as both *in vitro* aged cultures showed no matrix mineralization. In a complementary approach MSCs were differentiated into the osteogenic direction by OM supplementation with BMP2. Similarly to dexamethasone stimulation, BMP2 induced strong matrix mineralization in aMSCs_P2_ and yMSCs_P2_ but not in long-term cultured MSCs_P30_ and MSCs_P100_ (Figure 3B).

The adipogenic differentiation potential of the *in vitro* aged aMSCs_P30_ (mean_OR/AB_ = 0.28), yMSCs_P30_ (mean_OR/AB_ = 0.34), aMSCs_P100_ (mean_OR/AB_ = 0.30) and yMSCs_P100_ (mean_OR/AB_ = 0.29) was significantly decreased compared to aMSCs_P2_ (mean_OR/AB_ = 0.53, p_P2 vs. P30_ = 0.011, p_P2 vs. P100_ = 0.003) and yMSCs_P2_ (mean_AR/AB_ = 0.49, p_P2 vs. P30_ = 0.039, p_P2 vs. P100_ = 0.018), but remained significantly higher than the negative control cultured in EM (mean_OR/AB_ = 0.17) (Figure 3C). Likewise to osteogenic differentiation, no difference in adipogenic differentiation properties was observed between aMSCs and yMSCs at the same passage number.

Since it is known that primary aMSCs and yMSCs differ in their migration capacity [10], the migration potential of MSCs after long-term cultivation was assessed. With a modified Boyden chamber assay a significantly decreased migration rate of *in vitro* aged MSCs_P30_ and MSCs_P100_ compared to their primary counterparts was measured (Figure 3D). Moreover, the MSC migration potential declined significantly with the donor age (aMSCs vs. yMSCs: p_P2_ = 0.029, p_P30_ = 0.010, and p_P100_ = 0.031). Thus, our findings indicate an impact of both chronological and *in vitro* aging on MSC migratory capacity.

### Expression of genes associated with actin cytoskeleton organization and mitochondrial capacity is altered during *in vitro* MSC aging

**Figure 4:**
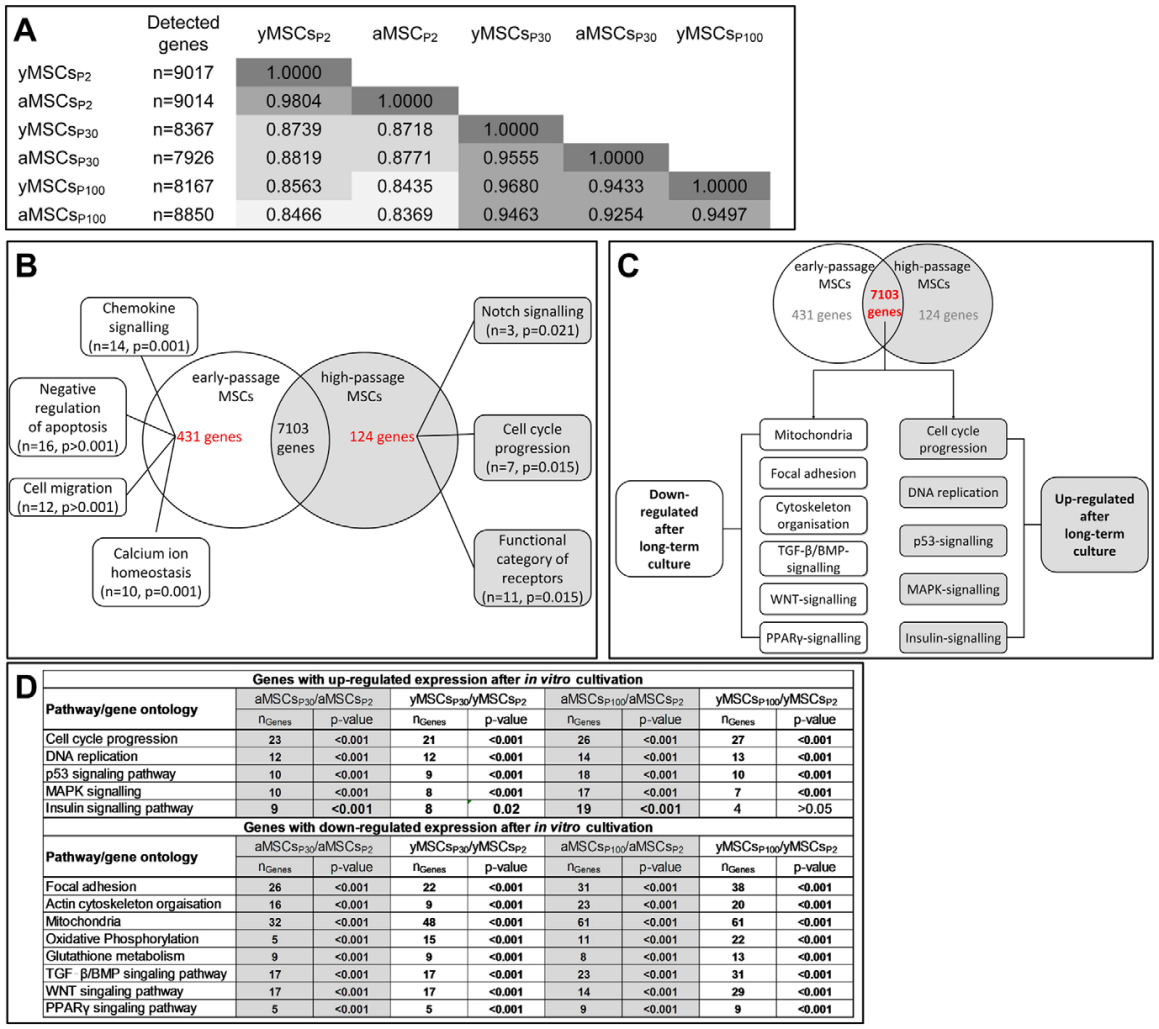
Transcriptional profiling of aMSCs and yMSCs at P2, P30 and P100. (**A**): The absolute number of genes detected after thresholding diminished during advanced *in vitro* culture independent from donor age (second column). The correlation coefficient (r^2^) was significantly reduced between aMSCs and yMSCs of P30 and P100 compared to P2. Only minor differences in gene expression were detected between aMSCs and yMSCs of each passage. (**B**): Functional annotation clustering of genes exclusively expressed either in primary MSC of P2 or *in vitro* aged MSCs of P30 and P100 revealed 431 and 124 differentially regulated genes, respectively. At P2 genes were mainly associated with chemokine signaling, apoptosis, cell migration, and calcium homeostasis. Whereas at P30 and P100 exclusively expressed genes are involved in Notch signaling, cell cycle progression and receptor signaling. (**C**): Analysis of pathways down-regulated after long-term *in vitro* culture revealed involvement of mitochondria, focal adhesions, cytoskeleton organization, TGF-β/BMP, WNT, and PPARγ signaling. Pathways up-regulated upon long-term *in vitro* culture were associated with cell cycle progression, DNA replication, p53, MAPK, and insulin signaling. (**D**): Differential statistical analysis summarizes all pathways and genes significantly up- and down-regulated during *in vitro* culture. The most numerous genes down-regulated during *in vitro* aging of aMSCs and yMSCs were associated with focal adhesions, actin cytoskeleton organization and mitochondrial function.

To supplement our functional analysis we compared the transcriptome of aMSCs and yMSCs at different *in vitro* passages with primary MSCs_P2_ cultures. Using Illumina® BeadArray technology, approximately 9000 genes were significantly detected in aMSCs_P2_ and yMSCs_P2_, while approximately 8000 genes were detected in each group of *in vitro* aged cells (Figure 4A). This observation indicates alterations of the expression profiles during increased cultivation time, which is supported by diminished correlation coefficients (r^2^) for MSCs_P30_ and MSCs_P100_ compared to MSCs_P2_ cultures. Functional annotation clustering revealed 431 genes specifically expressed in MSCs_P2_, 124 genes specifically expressed in long-term MSCs_P30&P100_ and 7103 genes that are expressed in either of them (Figure 4B). Pathway analysis detected in MSCs_P2_ chemokine signaling, negative regulation of apoptosis, cell migration, and calcium ion homeostasis as specific functional clusters (Table S3). In long-term MSCs_P30_ and MSCs_P100_ exclusively expressed genes clustered to Notch signaling, cell cycle progression and category of receptors (Table S4).

To further explore pathways that are specific for primary but not *in vitro* aged MSCs, we performed a detailed analysis of down- and up-regulated genes (detection p<0.01, expression ratio >1.5, differential p<0.05). In total 1199 mRNAs were differentially expressed between MSCs_P30_ and MSCs_P2_ (MSCs_P30_/MSCs_P2_: n_up-regulated_ = 460; n_down-regulated_ = 739). The expression of 1542 mRNAs was altered between MSCs_P100_ and MSCs_P2_ (MSCs_P100_/MSCs_P2_: n_up-regulated_ = 668; n_down-regulated_ = 874).

Functional annotation clustering of up-regulated genes upon long-term cultivation (Table S5) revealed an association of cell cycle progression, DNA replication, p53 signaling, and mitogen-activated protein kinase (MAPK) signaling with MSC *in vitro* aging (Figure 4C and D). Overall, these up-regulated pathways were more prominent in aMSCs_P100_ than in yMSCs_P100_. Genes involved in the insulin signaling pathway, were also up-regulated in long-term cultivated aMSCs, while only a minority of these genes were up-regulated in long-term cultivated yMSCs.

Annotation clustering of down-regulated genes upon *in vitro* aging (Table S6) revealed an association with focal adhesion function, actin cytoskeleton organization, TGF-β, WNT, PPARγ signaling, and mitochondrial capacity (Figure 4C and D). The down-regulation of genes with focal adhesion and actin cytoskeleton function is well consistent with our functional observations showing a reduced cell size, diminished cell spreading and decreased migration capacity of *in vitro* aged MSCs_P30_ and MSCs_P100_. Alterations in gene expression associated with the Tgf/Bmp signaling pathway, which is supported by impaired osteogenic differentiation of *in vitro* aged MSCs under BMP2 stimulation, was validated by quantitative real time RT-PCR. In accordance with trancriptome analysis, the expression of Bmpr1a, Bmpr2 and Bmp6 was down-regulated in MSCs_P100_ compared to primary MSCs_P2_ (Figure S3). In addition, aMSCs_P2_ displayed a significant higher expression level of Bmp6 compared to yMSCs_P2_. Moreover, Bmp2 expression was elevated in aMSCs_P100_, whereas it was reduced in yMSCs_P100_ compared to primary aMSCs_P2_ and yMSCs_P2_. Similarly, the expression of Bmpr1b was down-regulated in aMSCs_P100_, while it was up-regulated in yMSCs_P100_ compared to primary MSCs_P2_. Transcriptome analysis surprisingly demonstrated a specific impact of long-term *in vitro* culture on mitochondrial capacity, oxidative phosphorylation and glutathione metabolism of MSCs. This suggests a critical impact of energy metabolism for primary MSC function.

### The course of long-term cultivation alters mitochondria function

**Figure 5:**
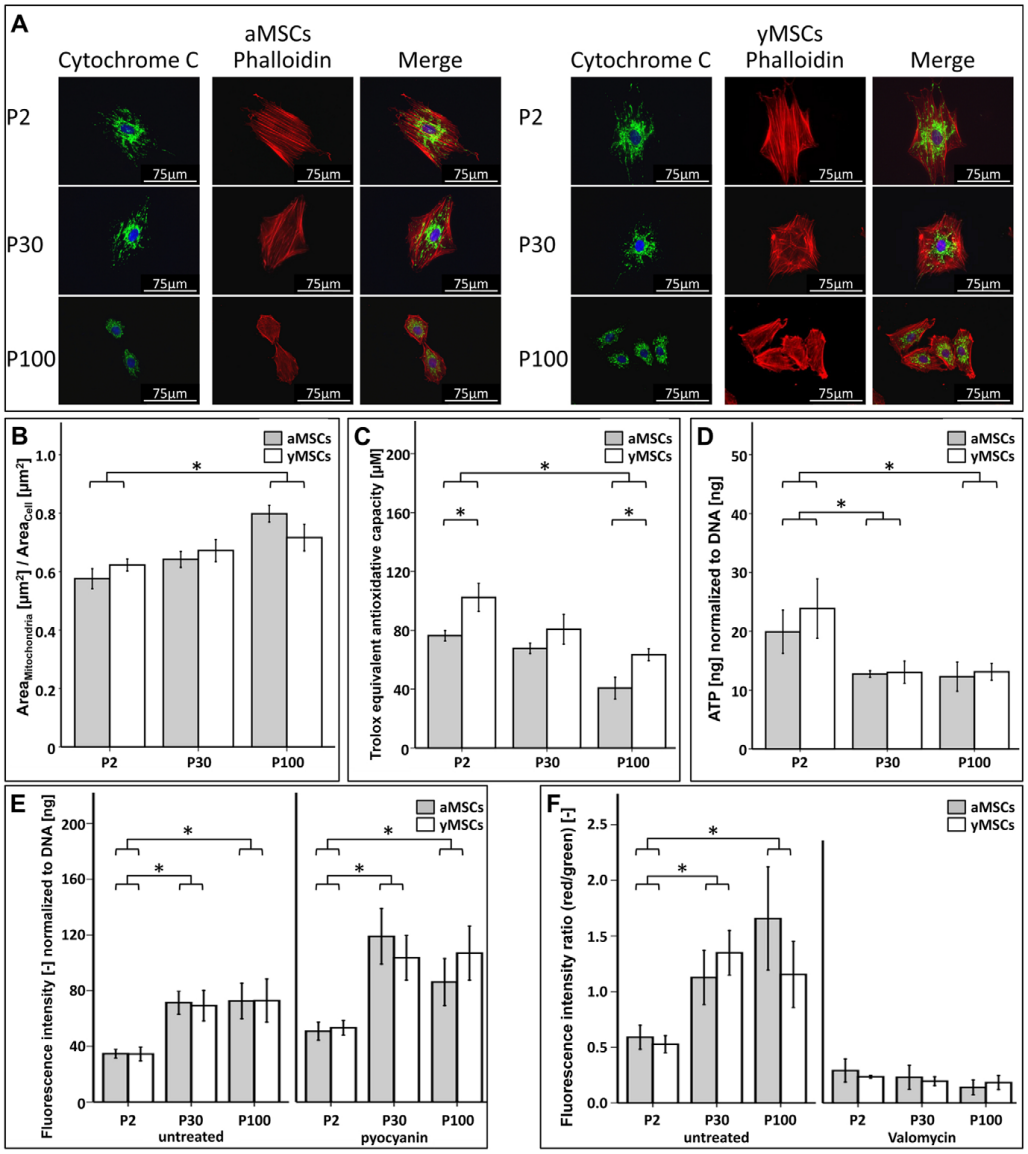
Long-term cultivation of MSCs alters their mitochondrial function. (**A**): Fluorescence microscopy was used to investigate the morphology of the mitochondrial network within long-term cultivated and primary aMSCs and yMSCs. Upon *in vitro* aging mitochondrial network appeared to be altered. Images show immunofluorescence of mitochondria and the actin cytoskeleton stained with a specific antibody recognizing cytochrome C and Alexa 594-conjugated phalloidin, respectively. Nuclei were counterstained with DAPI. (**B**): During *in vitro* aging the relative mitochondrial area per cell area increases in aMSCs and yMSCs of passage P30 and P100 compared to P2. The mitochondrial network and the cellular area were quantified after staining with MitoTracker™ Red and phalloidin, respectively. Diagram values represent ratio of the mitochondria network area relative to the cell area. (**C**): The total antioxidant capacity decreases with increasing passage number. Moreover, yMSCs of P2 and P100 exhibited significant higher antioxidant activities than aMSCs of the same passage. The Trolox® equivalent antioxidant assay kit was used to determine the total antioxidant capacity of whole MSC lysates and quantified against a Trolox® standard row. (**D**): Intracellular ATP levels decline significantly in long-term cultivated aMSCs and yMSCs of passage P30 and P100. Cellular ATP was determined using ATPLite™ bioluminescence luciferase-based assay and normalized to total DNA content determined by CyQuant®. (**E**): Long-term cultivated yMSCs and aMSCs of P30 and P100 displayed higher ROS production than primary MSCs of P2. After treatment with pyocyanin, which increases ROS levels, the observed difference between P2 and P30/P100 remained but the absolute value amplified about 2-fold. Intracellular ROS level were determined using CM-H2-DCFDA and normalized to total DNA content. (**F**): Measurement of the mitochondrial membrane potential (ΔΨm) revealed a progressive increase during *in vitro* aging with highest values in aMSCs of P100. Upon treatment with valomycin, an inhibitor of the mitochondrial respiratory chain, ΔΨm declined in aMSCs and yMSCs of all passages. The mitochondrial ΔΨm was determined with the MitoProbe® JC-1. * indicates statistical significance (p<0.05).

According to our ontology analysis, a major group of genes down-regulated upon *in vitro* aging is related to mitochondria. To further validate these *in silico* data functionally, the mitochondria network morphology was assessed (Figure 5A). During *in vitro* culture the ratio of mitochondria network area to the total cell area increases (Figure 5B).

We further measured the antioxidant power in lysates of *in vitro* aged MSCs using the Trolox® equivalent antioxidative capacity (TEAC) assay. With increased passage number a general reduction in antioxidant capacity was detected (aMSCs: TEAC_P2_ = 76 µM, TEAC_P30_ = 68 µM p = 1.000, TEAC_P100_ = 41 µM p = 0.001; yMSCs: TEAC_P2_ = 102 µM, TEAC_P30_ = 81 µM p = 0.035, TEAC_P100_ = 63 µM p<0.001) (Figure 5C). Moreover, the total antioxidant capacity in yMSCs was generally higher than in aMSCs at all different passages.

The mitochondrial function was further assessed by measuring the ATP content, production of intracellular ROS and the mitochondrial membrane potential (ΔΨm). Long-term cultivated MSCs_P30_ and MSCs_P100_ exhibited a significantly reduced content of cellular ATP than the primary MSCs_P2_ (aMSCs: ratio_P2_ = 19.1 nM/ng_DNA_, ratio_P30_ = 12.8 nM/ng_DNA_ p = 0.040, ratio_P100_ = 12.3 nM/ng_DNA_ p = 0.025; yMSCs: ratio_P2_ = 23.9 nM/ng_DNA_, ratio_P30_ = 13.0 nM/ng_DNA_ p = 0.001, ratio_P100_ = 13.1 nM/ng_DNA_ p = 0.001) (Figure 5D). Notably, no donor age-dependent difference in the ATP content was observed in the long-term cultivated as well as primary MSC populations.

Conversely to the decreased ATP content, intracellular ROS levels were found to be increased during *in vitro* aging (Figure 5E). Long term cultivated MSCs exhibited significant higher level of intracellular ROS compared to their primary counterparts (aMSCs: ratio_P2_ = 34.7FI/ng_DNA_, ratio_P30_ = 71.3FI/ng_DNA_ p = 0.002, ratio_P100_ = 72.6FI/ng_DNA_ p = 0.021; yMSCs: ratio_P2_ = 34.5FI/ng_DNA_, ratio_P30_ = 69.2FI/ng_DNA_ p = 0.006, ratio_P100_ = 72.8FI/ng_DNA_ p = 0.020). Under pyocyanin treatment, which induces intracellular ROS, all tested MSC population showed a significant increase of intracellular ROS level. No donor age-related differences were observed in the investigated MSC populations.

By measuring the ΔΨm with JC-1 a general increase during long-term cultivation was detected (aMSCs: ratio_P2_ = 0.6; ratio_P30_ = 1.1 p = 0.002; ratio_P100_ = 1.7 p<0.001; yMSCs: ratio_P2_ = 0.5; ratio_P30_ = 1.4 p<0.001; ratio_P100_ = 1.2 p = 0.029,) (Figure 5F). No statistical significant difference in ΔΨm was observed between aMSCs and yMSCs of the same passage. In summary, our data show that *in vitro* aging increases intracellular ROS levels, enhances ΔΨms and diminishes the intracellular ATP concentration.

## Discussion

Cell-based therapies utilizing MSCs have developed increasing importance for clinical application [20]. Limitations for clinical usage of MSCs are the lack of standardized isolation protocols, reliable quality control and sufficiently high cell quantities [21], [22]. Thus, to achieve adequate amounts of MSCs *in vitro* expansion is required [23]. Adversely, *in vitro* culture has a significant influence on gene expression and functional behavior of MSCs as soluble factors and cells forming the specific niche are lacking [24]. Cellular alterations occurring during *in vitro* aging were suggested to be similar to differences observed between MSCs from aged and young donors [14]. Hence, we here aimed to characterize the differential impact of donor animal age and *in vitro* aging for MSC function and gene expression.

### MSC *in vitro* aging and its implications for cell cycle progression, senescence and transformation

The proliferation capacity of the most cell types is limited. After a certain number of cell divisions, the population expansion is slowed down, before the cells ultimately stop dividing [25]. Moreover, it is assumed that the number of cell division cycles decreases with the donor age. In our study, we observed no ultimate cell cycle arrest of the entire cell population during *in vitro* aging of aMSCs and yMSCs from rats. Culture of aMSCs and yMSCs occurred for more than 100 passages at relative constant proliferation rates, suggesting extension of lifespan and possibly spontaneous immortalization. Extended long-term culture might result in spontaneous immortalization of murine as well as human MSCs [13], [26], [27]. In contrast to other studies, neither aMSCs_P100_ nor yMSCs_P100_ showed growth in a soft agar assay suggesting an untransformed status [28]. Notably, a non-transformed status does not exclude the occurrence of aneuploidy in long-term cultivated MSCs. Other groups have shown that MSCs with chromosomal instabilities exhibited no evidence of transformation either *in vitro* or *in vivo* and enter senescence [29]. Accordingly, transcriptional analysis revealed an up-regulation of genes associated with the tumor suppressor p53 signaling pathway. This pathway promotes replicative and premature senescence as well as apoptosis [30], [31]. It seems to be progressively activated during aging in response to various cellular stresses, including DNA damage and oncogene activation [32]. Correspondingly, p53 signaling is inactive in the majority of human cancer cells and partially accounts for their resistance to senescence [30]. Thus, *in vitro* aging induces increased p21^WAF1/CIP1^ and p16^INK4A^ expression pointing to a higher proportion of senescent cells in long-term cultivated MSCs.

It is important to note that increased expression of senescence markers in long-term cultivated MSC population does not necessarily indicate a decline in replicative potential of each individual cell. Rather to replicative senescence, a proportion of individual cells might undergo stress-induced premature senescence in response to intracellular stress like oxidative stress, irreversible DNA damage or genomic instability [30], [33]. For example, it was shown that both chemical and culture-induced oxidative stress cause DNA damages and aneuploidy in human MSCs, which subsequently undergo senescence [34].

The loss of cells due to senescence might be counterbalanced by other fast growing cells, marked by the up-regulation of genes associated the cell cycle progression, which explains the unaltered proliferation rate of the whole *in vitro* aged MSC populations in comparison to the primary MSC cultures.

### *In vitro* aging negatively affects the differentiation capacity of aMSCs and yMSCs

Long-term culture not only affects aMSCs and yMSCs on a molecular level; it also alters their morphology and has functional consequences. MSCs of P30 and P100 were no longer able to differentiate into the osteogenic lineage and the ability to differentiate into the adipogenic lineage was markedly decreased. These results are consistent with several other studies showing a reduced differentiation potential in human and murine MSCs upon *in vitro* aging [35]–[38]. The mRNA expression analysis revealed a decreased expression of genes involved in Wnt and Tgf-β/Bmp signaling upon long term culture. The Wnt pathway is clearly required throughout osteogenesis and substantial elevated Wnt/β-catenin signaling triggers the differentiation of MSCs into the osteogenic lineage [39]–[42].

A pivotal role of Bmp signaling is the induction of bone and cartilage formation [43]. BMPs induce the differentiation of mesenchymal cells and also enhance the function of osteoblasts (matrix synthesis) [44]. Some studies have pointed out that BMP signaling is also required for adipogenic differentiation of mesenchymal precursor cells [44]. Accordingly, we observed decreased adipogenic differentiation potentials of long-term cultivated MSCs and a down-regulation of genes involved in the PPARγ pathway. The PPARγ pathway positively regulates the adipocytic differentiation of MSCs and intracellular accumulation of lipids by modulating genes involved in their uptake and metabolism [45].

Notably, the BMP- as well as dexamethasone induced osteogenic differentiation was impaired in long-term cultivated MSCs. Also others have reported that a decreased differentiation potential upon dexamethasone-induced osteogenic differentiation is associated with altered Bmp-receptor mRNA expression [44]–[46]. This observation emphasizes the important role of down-regulated BMP signaling for the diminished differentiation potential of *in vitro* aged MSCs. Results from several studies have led to the assumption that the BMP pathway cooperates with other pathways, especially the canonical Wnt-signaling [46]–[48], to drive osteogenic differentiation. For example, the knock-out of the Wnt/ß-catenin antagonist Axin2 leads to enhanced nuclear accumulation of ß-catenin and increased levels of BMP2, BMP6 and phosphor-Smad, which further promotes osteogenic differentiation of osteoprogenitor cells and enhances bone formation *in vitro* and *in vivo*, respectively [49], [50]. In both studies, the effect of the Axin2 knockout on BMP-signaling and osteogenic differentiation could be reversed by ß-catenin inactivation. Collectively, there is growing evidence suggesting that osteogenic differentiation of osteoprogenitor cells is highly dependent on the cross talk between Wnt and BMP signaling [46]. Moreover, aMSCs and yMSCs exhibit exclusive expression of several genes belonging to the Notch signaling pathway upon *in vitro* aging. The Notch signaling pathway is known to suppress osteogenic differentiation and markedly decreases trabecular bone mass in adolescent mice [51]. Thus, the observed alterations in Tgf/Bmp-, Wnt-, Pparγ-, Mapk- and Notch signaling pathway might act together and lead to the loss of differentiation ability after long-term culture. Altogether, transcriptomes of *in vitro* aged aMSCs and yMSCs are clearly distinct from primary MSCs (approx. 85% correlation); therefore a complete loss of progenitor characteristic with long-term cultivation is reasonable to expect. In conclusion, long-term survival of MSCs in culture is achieved at the cost of differentiation potential. Furthermore, *in vitro* cell culture conditions favor expansion of cells with high proliferation potential rather than those with high differentiation potential. Here we have shown that *in vitro* aged MSCs express common markers of the MSCs phenotype, which highlights the lack of reliable markers for multipotent MSCs.

### Altered morphology and migration potential upon *in vitro* aging

Apart from the negative influence of *in vitro* aging on MSC differentiation potential, extended long-term culture might also lead to other functional alterations. For instance, recently we and others have shown that chronological aging has a significant impact on cell migration, cytoskeleton organization and actin turn-over [10], [52]. As MSCs for each therapeutic approach crucially rely on proper migration towards stimuli for functional engraftment, we analyzed aMSCs and yMSCs for this aspect in more detail. Random (undirected) migration potential of MSCs in our study was affected by both *in vitro* aging status and age of donor animals. Cellular migration, which requires coordinated contact to the extracellular substrate followed by detachment, strongly depends on local cytoskeleton organization and actin turn-over [53]. The impact of local actin organization for migration is accented by the importance of lamellipodia, fillopodia and focal complex formation for cellular migration [54]. In line with the reduced MSC migration potential upon in vitro and chronological aging, diminished expression of genes associated with focal adhesion and actin cytoskeleton organization was observed. Differential gene regulation was more prominent between primary and long-term cultivated cells than between aMSCs and yMSCs of the same passage as reflected by a higher number of affected genes, higher degree of differential expression as well as lower p-values. By immunofluorescence analysis, we demonstrated that during long-term cultivation size of aMSCs and yMSCs as well as their filopodia and lamellipodia number decreases, while their cell roundness increases. Correspondingly, with increasing passage number we observed a donor-age independent decline in mRNA expression of specific genes subsets encoding cytoskeletal and focal adhesion proteins such as integrins, alpha-actinins, actin related protein 2/3 complex, Rho-associated coiled-coil forming kinases (ROCK), cofilin, and profilin.

Although undirected transmigration rather than chemotaxis was investigated, it needs to be highlighted that we further observed a down-regulation of mRNAs of several chemokines, cytokines and their receptors during chronological and *in vitro* aging, e.g. stromal cell-derived factor 1 (Sdf-1) and its receptor (Cxcr4). On molecular level, it is assumed that specific chemokines and their receptors play a critical role to direct MSCs to their desired site of action. Studies investigating skeletal repair and systemic skeletal disorders in animal models showed that CXCR4 and SDF-1 recruit MSCs to the fracture site and prevent bone loss [55].

### Oxidative stress might cause the decline of MSC functionality upon *in vitro* aging

Transcriptome analysis revealed a passage-dependent decline in the expression of mRNA associated with mitochondria, oxidative phosphorylation, glutathione metabolism, and antioxidant defense. These expression changes were accompanied by altered mitochondria morphology, reduced antioxidant capacity, increased ROS levels, enhanced ΔΨms, and diminished intracellular ATP concentrations. Dysfunctional mitochondria, which are a natural source of free radicals and ROS, lead to increased intracellular ROS concentration, impaired ATP production and causes stress-induced senescence in normal somatic cells as well as in MSCs [56]–[58]. This together suggests that the higher expression of senescence markers in long-term cultured MSCs may be caused by increased intracellular stress. In human ESCs and iPSCs alterations in mitochondrial proliferation and development were associated with loss of pluripotency [18]. ESCs and iPSCs that demonstrate reduced mitochondrial number and activity suppress the mitochondrial/oxidative stress pathway. Moreover, iPSCs exhibit alterations of senescence-related p53 signaling pathway compared to their differentiated and subsequently transformed cellular origin. Furthermore, long-term cultured hESCs are characterized by dysfunctional mitochondria potentially compromising their long-term pluripotency [59]. Similar to our observations, the same study associated an elevated mitochondria network volume with increased ΔΨm and ROS levels. These changes were attributed to a diminished removal of damaged mitochondria and/or fusion of existing mitochondria in order to compensate for mitochondrial dysfunction. Although chronological aging also affects MSCs antioxidant capacity and glutathione metabolism, we find no difference in the basal ROS levels comparing aMSCs and yMSCs. This observation is in line with other studies demonstrating unaffected ROS production in cells from aged and young adult rats [60]. Thus, our findings suggest that increased intracellular oxidative stress could be the basis for the progressive functional decline of aMSCs and yMSCs during long-term *in vitro* culture. Mitochondrial dysfunction marked by increased ROS concentrations may lead to DNA and protein damage, which in turn might activates p53 signaling increasing the amount of senescent and dysfunctional cells. These detrimental effects seem to occur independently of the donor age during *in vitro* expansion and support the idea that chronological and *in vitro* aging are distinct processes.

## Conclusion

Long-term *in vitro* culture, but not chronological aging, compromises the osteogenic and adipogenic differentiation capacity of MSCs and alters their morphology, susceptibility to senescence and mitochondrial function. Accordingly, transcriptome analysis revealed that chronological and *in vitro* aging results to a large extent in divergent changes at the molecular level. Thus, independent from donor animal age, *in vitro* aging of MSCs seems to result in complete loss of their progenitor characteristics. Although, *in vitro* aging alters the migration potential and antioxidative capacity of MSCs as a function of the donor age, results of this study collectively suggest that both are distinct processes. Even if our present study is in some way limited by the usage of rat MSCs instead of human MSCs, it provides direct comparison between *in vitro* and chronological aged MSC not only at the cellular but also at the molecular level. Perspectively, therapeutic approaches utilizing MSCs should critically review *in vitro* expansion.

## Supporting Information

Figure S1 **Cell roundness increases during long term culture of aMSCs and yMSCs.** Actin fibers were stained with Alexa 594-conjugated phalloidin (6.6 nM). Nuclei were counterstained with DAPI. Fluorescence images were taken under identical excitation and exposure conditions. Cell roundness were quantified using Columbus 2.0 software (PerkinElmer) and results are presented as mean ± standard error of the mean (SEM). Abbreviations: aMSCs, mesenchymal stromal cells from aged donors; yMSCs, mesenchymal stromal cells from young donors; P, passage; * indicates statistical significance (p<0.05).
(TIF)

Figure S2 **Cell surface marker pattern of long-term cultivated aMSCs and yMSCs of passage 100.** MSC phenotype was characterized by flow cytometry (n = 3). Representative pictures are shown: Both aMSCs and yMSCs of passage 100 were positive for CD29, CD44, CD73, CD90, CD105, CD106, CD166 and RT1A as well as negative for CD45, CD34 and RT1B. Abbreviations: aMSCs, mesenchymal stromal cells from aged donors in passage 100; yMSCs, mesenchymal stromal cells from young donors in passage 100.
(TIF)

Figure 3 **Down-regulation of Bmpr1a, Bmpr1b and Bmpr2 as well as Bmp6 after long-term cultivation.** Diagrams show mRNA expression levels of BmpR1a, BmpR1b, BmpR2, Bmp6 and Bmp2 normalized to Eef1a. Abbreviations: aMSCs, mesenchymal stromal cells from aged donors; yMSCs, mesenchymal stromal cells from young donors; eEf1a, Elongation factor 1-alpha; BmpR1a, Bone morphogenetic protein receptor type-1a; BmpR1b, Bone morphogenetic protein receptor type-1b; BmpR2, Bone morphogenetic protein receptor type-2; Bmp6, Bone morphogenetic protein 6. (n = 3) * indicates statistical significance (p<0.05).
(TIF)

Table S1 **Antibodies used for flow cytometry.**
(DOC)

Table S2 **Primer sequences used for quantitative RT-PCR.**
(DOC)

Table S3 **Exclusively expressed mRNAs in primary MSCs of passage 2.**
(DOC)

Table S4 **Exclusively expressed mRNAs in long-term cultivated MSCs of passage 30 and 100.**
(DOC)

Table S5 **Genes with up-regulated expression after long-term cultivation.**
(DOC)

Table S6 **Genes with down-regulated expression after long-term cultivation.**
(DOC)
