# Supplementary figures and images for: Functional Comparison of Chronological and In Vitro Aging: Differential Role of the Cytoskeleton and Mitochondria in Mesenchymal Stromal Cells
Source: PLoS One. 2012 Dec 28;7(12):e52700. doi: 10.1371/journal.pone.0052700 (PMC3532360; doi:10.1371/journal.pone.0052700)

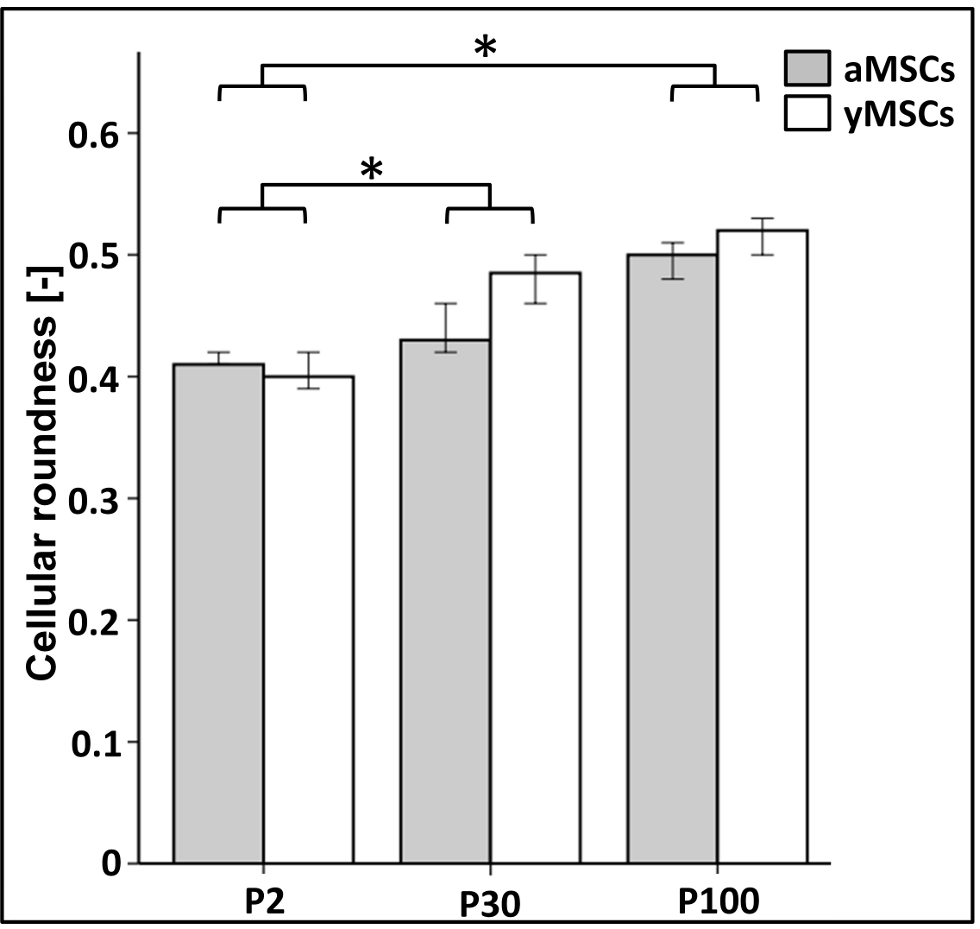

Supplement: Figure S1 — Cell roundness increases during long term culture of aMSCs and yMSCs. Actin fibers were stained with Alexa 594-conjugated phalloidin (6.6 nM). Nuclei were counterstained with DAPI. Fluorescence images were taken under identical excitation and exposure conditions. Cell roundness were quantified using Columbus 2.0 software (PerkinElmer) and results are presented as mean ± standard error of the mean (SEM). Abbreviations: aMSCs, mesenchymal stromal cells from aged donors; yMSCs, mesenchymal stromal cells from young donors; P, passage; * indicates statistical significance (p<0.05). (TIF) [file pone.0052700.s001.tif]

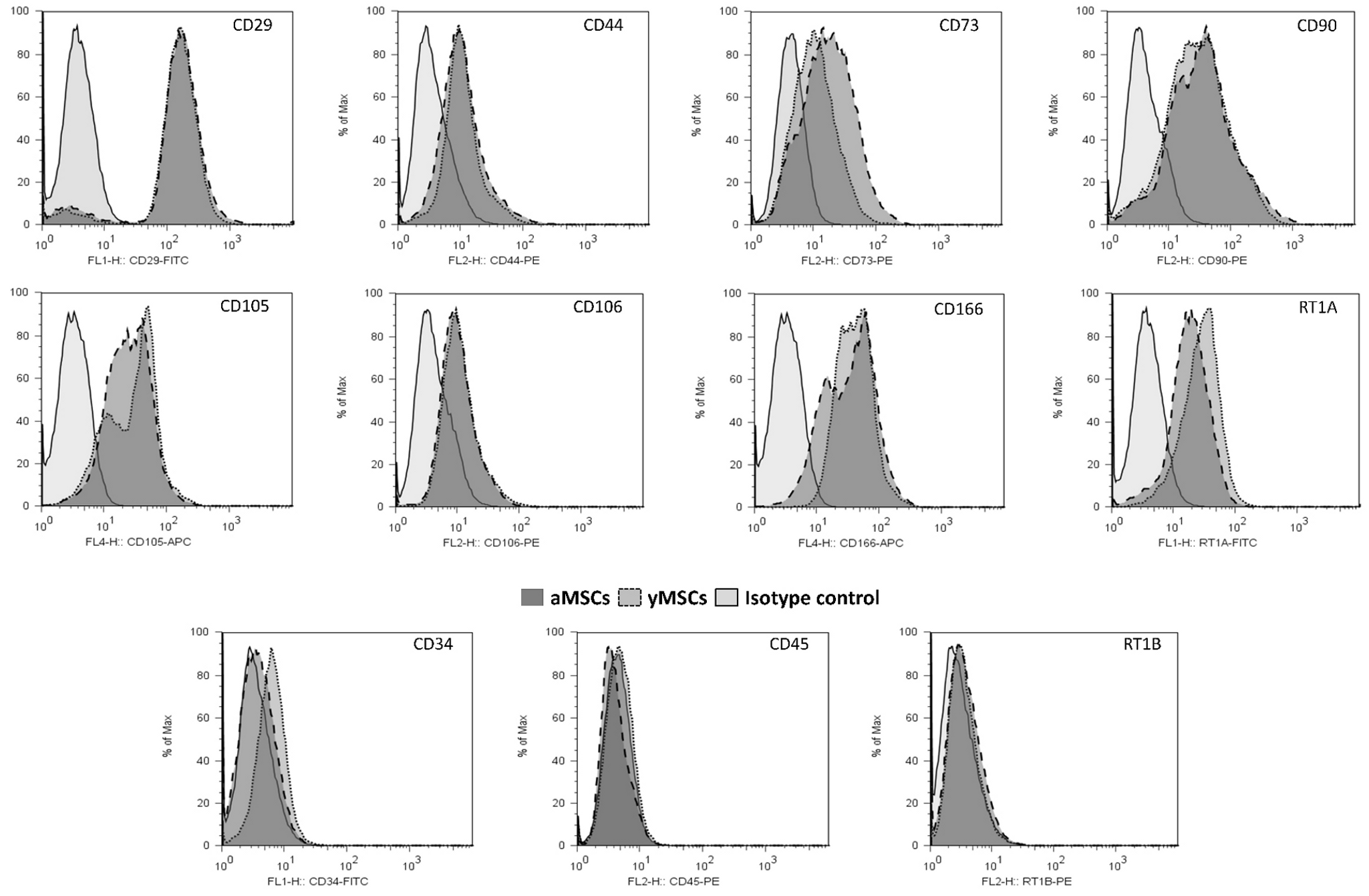

Supplement: Figure S2 — Cell surface marker pattern of long-term cultivated aMSCs and yMSCs of passage 100. MSC phenotype was characterized by flow cytometry (n = 3). Representative pictures are shown: Both aMSCs and yMSCs of passage 100 were positive for CD29, CD44, CD73, CD90, CD105, CD106, CD166 and RT1A as well as negative for CD45, CD34 and RT1B. Abbreviations: aMSCs, mesenchymal stromal cells from aged donors in passage 100; yMSCs, mesenchymal stromal cells from young donors in passage 100. (TIF) [file pone.0052700.s002.tif]

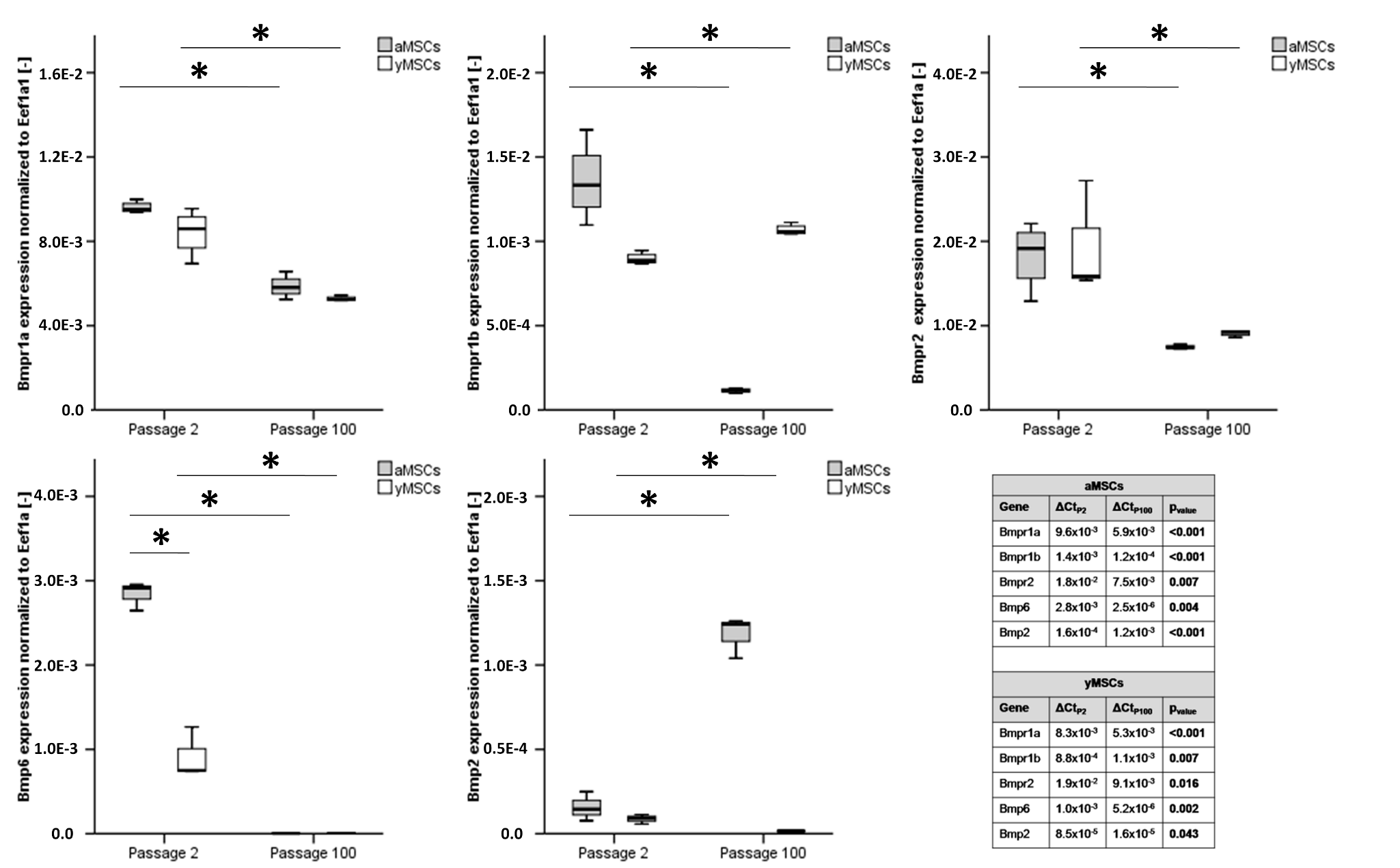

Supplement: Figure 3 — Down-regulation of Bmpr1a, Bmpr1b and Bmpr2 as well as Bmp6 after long-term cultivation. Diagrams show mRNA expression levels of BmpR1a, BmpR1b, BmpR2, Bmp6 and Bmp2 normalized to Eef1a. Abbreviations: aMSCs, mesenchymal stromal cells from aged donors; yMSCs, mesenchymal stromal cells from young donors; eEf1a, Elongation factor 1-alpha; BmpR1a, Bone morphogenetic protein receptor type-1a; BmpR1b, Bone morphogenetic protein receptor type-1b; BmpR2, Bone morphogenetic protein receptor type-2; Bmp6, Bone morphogenetic protein 6. (n = 3) * indicates statistical significance (p<0.05). (TIF) [file pone.0052700.s003.tif]
