# Supplementary material for: Functional Comparison of Chronological and In Vitro Aging: Differential Role of the Cytoskeleton and Mitochondria in Mesenchymal Stromal Cells
Source: PLoS One. 2012 Dec 28;7(12):e52700. doi: 10.1371/journal.pone.0052700 (PMC3532360; doi:10.1371/journal.pone.0052700)
Supplement: Table S1 — Antibodies used for flow cytometry. (DOC) [file pone.0052700.s004.doc]

| **Table S1: Antibodies used for flow cytometry** | | |
| --- | --- | --- |
|  |  |  |
| **Target** | **Antibody** | **Supplier** |
| CD29 | hamster(α-rat CD29) | BD Biosciences, Germany, www.bdbiosciences.com |
| CD73 | mouse(α-rat CD73) | BD Biosciences, Germany, www.bdbiosciences.com |
| RT1A | mouse(α-rat RT1A [MHC class I]):FITC | BD Biosciences, Germany, www.bdbiosciences.com |
| RT1B | mouse(α-rat RT1B [MHC class II]):PE | BD Biosciences, Germany, www.bdbiosciences.com |
| CD45 | mouse(α-rat CD45) | Acris Antibodies, Germany, www.acris-antibodies.com |
| CD90 | mouse(α-rat CD90) | Acris Antibodies, Germany, www.acris-antibodies.com |
| CD44 | mouse(α-rat CD44) | AbD Serotec, UK, www.abdserotec.com |
| CD34 | mouse(α-rat CD34):FITC | Santa Cruz Biotechnology, Germany, www.scbt.com |
| CD105 | mouse(α-rat CD105) | Santa Cruz Biotechnology, Germany, www.scbt.com |
| CD166 | rabbit(α-rat CD166) | Biolegend, Germany, www.biolegend.com |
| - | isotype Armenian hamster IgG:FITC | Biolegend, Germany, www.biolegend.com |
| - | rat (α-mouse IgG):PE | BD Biosciences, Germany, www.bdbiosciences.com |
| - | donkey (α-rabbit IgG):Cy5 | Dianova, Germany |
