# Supplementary material for: Functional Comparison of Chronological and In Vitro Aging: Differential Role of the Cytoskeleton and Mitochondria in Mesenchymal Stromal Cells
Source: PLoS One. 2012 Dec 28;7(12):e52700. doi: 10.1371/journal.pone.0052700 (PMC3532360; doi:10.1371/journal.pone.0052700)
Supplement: Table S2 — Primer sequences used for quantitative RT-PCR. (DOC) [file pone.0052700.s005.doc]

| **Table S2: Primer sequences used for quantitative RT-PCR** | |  |
| --- | --- | --- |
|  |  |  |
| **Gene Symbol** | **Forward** | **Reverse** |
| *BmpR1a* | AGCCCTACATCATGGCTGAC | CTTCAAAACGGCTCGAAGAC |
| *BmpR1b* | TTGATTGAGCAATCGCAGAG | CAGAATGTTCTCGTGCCTCA |
| *BmpR2* | ACAAGACCTTGGGATTGGTG | TGAGGGTGGGGTGGTAGTTA |
| *Bmp6* | TAGCAATCTGTGGGTGGTGA | ACCTCGCTCACCTTGAAGAA |
| *Gapdh* | ATGGGAAGCTGGTCATCAAC | GTGGTTCACACCCATCACAA |
| *Actb* | TGTCACCAACTGGGACGATA | GGGGTGTTGAAGGTCTCAAA |
| *Eef1a* | CCCTGTGGAAGTTTGAGACC | CTGCCCGTTCTTGGAGATAC |
