# Supplementary material for: Functional Comparison of Chronological and In Vitro Aging: Differential Role of the Cytoskeleton and Mitochondria in Mesenchymal Stromal Cells
Source: PLoS One. 2012 Dec 28;7(12):e52700. doi: 10.1371/journal.pone.0052700 (PMC3532360; doi:10.1371/journal.pone.0052700)
Supplement: Table S3 — Exclusively expressed mRNAs in primary MSCs of passage 2. (DOC) [file pone.0052700.s006.doc]

**Table S3: Exclusively expressed mRNAs in primary MSCs of passage 2**

| **Chemokine signaling pathway (p=0.001)** | |  |  |  |  |  |  |
| --- | --- | --- | --- | --- | --- | --- | --- |
| ACCESSION | Name | yMSCs Passage 2 | | aMSCs Passage 2 | | Ratio | |
|  |  | AVG_Signal | P_Detection_ | AVG_Signal | P_Detection_ | aMSC/yMSCs | p_aMSCs vs. yMSCs_ |
| NM_024145 | Gardner-Rasheed feline sarcoma viral (Fgr) oncogene homolog (Fgr), mRNA. | 26.4 | **0.006** | 18.9 | **0.007** | 0.716 | 0.537 |
| NM_031007 | adenylate cyclase 2 (Adcy2), mRNA. | 119.9 | **0.000** | 69.8 | **0.001** | **0.582** | **0.001** |
| XM_226333 | PREDICTED: adenylate cyclase 7 (Adcy7), mRNA. | 50.1 | **0.002** | 34.0 | **0.002** | **0.679** | 0.210 |
| NM_031530 | chemokine (C-C motif) ligand 2 (Ccl2), mRNA. | 932.5 | **0.000** | 3208.1 | **0.000** | **3.440** | 0.373 |
| NM_019233 | chemokine (C-C motif) ligand 20 (Ccl20), mRNA. | 51.0 | **0.002** | 407.3 | **0.000** | **7.990** | 0.386 |
| NM_013025 | chemokine (C-C motif) ligand 3 (Ccl3), mRNA. | 28.5 | **0.005** | 48.4 | **0.002** | **1.696** | 0.673 |
| NM_001007612 | chemokine (C-C motif) ligand 7 (Ccl7), mRNA. | 486.8 | **0.000** | 2365.3 | **0.000** | **4.859** | 0.380 |
| NM_022205 | chemokine (C-X-C motif) receptor 4 (Cxcr4), mRNA. | 95.0 | **0.000** | 22.3 | **0.006** | **0.235** | **0.036** |
| NM_053734 | neutrophil cytosolic factor 1 (Ncf1), mRNA. | 162.3 | **0.000** | 101.8 | **0.000** | **0.627** | 0.502 |
| NM_001007729 | platelet factor 4 (Pf4), mRNA. | 603.8 | **0.000** | 92.9 | **0.000** | **0.154** | 0.075 |
| NM_012713 | protein kinase C, beta 1 (Prkcb1), mRNA. | 81.7 | **0.001** | 52.0 | **0.002** | **0.637** | 0.562 |
|  |  |  |  |  |  |  |  |
| **Chemokine-Chemokine receptor interaction** | |  |  |  |  |  |  |
| ACCESSION | Name | yMSCs Passage 2 | | aMSCs Passage 2 | | Ratio | |
|  |  | AVG_Signal | P_Detection_ | AVG_Signal | P_Detection_ | aMSC/yMSCs | p_aMSCs vs. yMSCs_ |
| NM_031530 | chemokine (C-C motif) ligand 2 (Ccl2), mRNA. | 932.5 | **0.000** | 3208.1 | **0.000** | **3.440** | 0.373 |
| NM_013025 | chemokine (C-C motif) ligand 3 (Ccl3), mRNA. | 28.5 | **0.005** | 48.4 | **0.002** | **1.696** | 0.673 |
| NM_022205 | chemokine (C-X-C motif) receptor 4 (Cxcr4), mRNA. | 95.0 | **0.000** | 22.3 | **0.006** | **0.235** | 0.066 |
| NM_031512 | interleukin 1 beta (Il1b), mRNA. | 64.1 | **0.002** | 109.5 | **0.000** | **1.709** | 0.676 |
| NM_019165 | interleukin 18 (Il18), mRNA. | 52.4 | **0.002** | 53.2 | **0.002** | 1.014 | 0.986 |
| NM_001005384 | oncostatin M specific receptor (Osmr), mRNA. | 54.6 | **0.002** | 61.4 | **0.002** | 1.123 | 0.639 |
| NM_001007729 | platelet factor 4 (Pf4), mRNA. | 603.8 | **0.000** | 92.9 | **0.000** | **0.154** | 0.075 |
| **Negative regulation of apoptosis (p>0.001)** | |  |  |  |  |  |  |
| ACCESSION | Name | yMSCs Passage 2 | | aMSCs Passage 2 | | Ratio | |
|  |  | AVG_Signal | P_Detection_ | AVG_Signal | P_Detection_ | aMSC/yMSCs | p_aMSCs vs. yMSCs_ |
| NM_133416 | Rattus norvegicus B-cell leukemia/lymphoma 2 related protein A1 (Bcl2a1), mRNA. | 142.6 | **0.000** | 46.8 | **0.002** | **0.328** | 0.145 |
| NM_017196 | Rattus norvegicus allograft inflammatory factor 1 (Aif1), mRNA. | 665.7 | **0.000** | 387.0 | **0.000** | **0.581** | 0.334 |
| NM_138828 | Rattus norvegicus apolipoprotein E (Apoe), mRNA. | 2431.9 | **0.000** | 331.2 | **0.000** | **0.136** | 0.089 |
| NM_001009626 | Rattus norvegicus apolipoprotein H (Apoh), mRNA. | 25.2 | **0.006** | 21.2 | **0.006** | 0.840 | 0.636 |
| NM_023987 | Rattus norvegicus baculoviral IAP repeat-containing 3 (Birc3), mRNA. | 23.8 | **0.007** | 33.5 | **0.002** | 1.410 | 0.521 |
| XM_214551 | PREDICTED: Rattus norvegicus cell death-inducing DNA fragmentation factor, alpha subunit-like effector A (predicted) (Cidea_predicted), mRNA. | 46.4 | **0.004** | 47.9 | **0.002** | 1.031 | 0.898 |
| NM_031530 | Rattus norvegicus chemokine (C-C motif) ligand 2 (Ccl2), mRNA. | 932.5 | **0.000** | 3208.1 | **0.000** | **3.440** | 0.373 |
| NM_017333 | Rattus norvegicus endothelin receptor type B (Ednrb), mRNA. | 62.8 | **0.002** | 68.9 | **0.002** | 1.097 | 0.746 |
| NM_031512 | Rattus norvegicus interleukin 1 beta (Il1b), mRNA. | 64.1 | **0.002** | 109.5 | **0.000** | **1.709** | 0.676 |
| XM_232855 | PREDICTED: Rattus norvegicus mitogen activated protein kinase kinase kinase 7 (predicted) (Map3k7_predicted), mRNA. | 20.2 | **0.008** | 21.3 | **0.006** | 1.056 | 0.857 |
| XM_574821 | PREDICTED: Rattus norvegicus similar to MADS box transcription enhancer factor 2, polypeptide C (myocyte enhancer factor 2C) (LOC499497), mRNA. | 28.0 | **0.006** | 20.2 | **0.006** | 0.723 | 0.313 |
| NM_019328 | Rattus norvegicus nuclear receptor subfamily 4, group A, member 2 (Nr4a2), mRNA. | 37.5 | **0.004** | 33.1 | **0.002** | 0.884 | 0.581 |
| NM_001007729 | Rattus norvegicus platelet factor 4 (Pf4), mRNA. | 603.8 | **0.000** | 92.9 | **0.000** | **0.154** | 0.075 |
| XM_342223 | PREDICTED: Rattus norvegicus protein kinase C, lambda (Pkcl), mRNA. | 26.3 | **0.006** | 23.8 | **0.005** | 0.905 | 0.768 |
| NM_022507 | Rattus norvegicus protein kinase C, zeta (Prkcz), mRNA. | 51.9 | **0.002** | 28.1 | **0.002** | **0.542** | **0.027** |
| NM_017059 | Rattus norvegicus Bcl2-associated X protein (Bax), mRNA. | 24.4 | **0.007** | 23.6 | **0.005** | 0.966 | 0.905 |
| **Cell migration (p=0.001)** | |  |  |  |  |  |  |
| ACCESSION | Name | yMSCs Passage 2 | | aMSCs Passage 2 | | Ratio | |
|  |  | AVG_Signal | P_Detection_ | AVG_Signal | P_Detection_ | aMSC/yMSCs | p_aMSCs vs. yMSCs_ |
| XM_217246 | PREDICTED: non-catalytic region of tyrosine kinase adaptor protein 1 (predicted) (Nck1_predicted), mRNA. | 20.9 | **0.008** | 17.9 | **0.008** | 0.854 | 0.619 |
| NM_031530 | chemokine (C-C motif) ligand 2 (Ccl2), mRNA. | 932.5 | **0.000** | 3208.1 | **0.000** | **3.440** | 0.373 |
| NM_013025 | chemokine (C-C motif) ligand 3 (Ccl3), mRNA. | 28.5 | **0.005** | 48.4 | **0.002** | **1.696** | 0.673 |
| NM_022205 | chemokine (C-X-C motif) receptor 4 (Cxcr4), mRNA. | 95.0 | **0.000** | 22.3 | **0.006** | **0.235** | 0.066 |
| NM_172333 | collagen triple helix repeat containing 1 (Cthrc1), mRNA. | 208.7 | **0.000** | 39.9 | **0.002** | **0.191** | **0.000** |
| NM_130411 | coronin, actin binding protein 1A (Coro1a), mRNA. | 174.8 | **0.000** | 119.9 | **0.000** | 0.686 | 0.507 |
| NM_017333 | endothelin receptor type B (Ednrb), mRNA. | 62.8 | **0.002** | 68.9 | **0.002** | 1.097 | 0.746 |
| NM_012951 | fibroblast growth factor 10 (Fgf10), mRNA. | 28.1 | **0.006** | 39.5 | **0.002** | 1.404 | 0.198 |
| NM_031512 | interleukin 1 beta (Il1b), mRNA. | 64.1 | **0.002** | 109.5 | **0.000** | **1.709** | 0.676 |
| NM_019328 | nuclear receptor subfamily 4, group A, member 2 (Nr4a2), mRNA. | 37.5 | **0.004** | 33.1 | **0.002** | 0.884 | 0.581 |
| NM_001007729 | platelet factor 4 (Pf4), mRNA. | 603.8 | **0.000** | 92.9 | **0.000** | **0.154** | 0.075 |
| NM_017059 | Bcl2-associated X protein (Bax), mRNA. | 24.4 | **0.007** | 23.6 | **0.005** | 0.966 | 0.905 |
| **Calcium ion homeostasis (p=0.001)** | |  |  |  |  |  |  |
| ACCESSION | Name | yMSCs Passage 2 | | aMSCs Passage 2 | | Ratio | |
|  |  | AVG_Signal | P_Detection_ | AVG_Signal | P_Detection_ | aMSC/yMSCs | p_aMSCs vs. yMSCs_ |
| NM_031648 | Rattus norvegicus FXYD domain-containing ion transport regulator 1 (Fxyd1), mRNA. | 53.9 | **0.002** | 42.7 | **0.002** | 0.793 | 0.275 |
| NM_030985 | Rattus norvegicus angiotensin II receptor, type 1 (AT1A) (Agtr1a), mRNA. | 96.7 | **0.000** | 40.2 | **0.002** | **0.416** | **0.000** |
| NM_138828 | Rattus norvegicus apolipoprotein E (Apoe), mRNA. | 2431.9 | **0.000** | 331.2 | **0.000** | **0.136** | 0.089 |
| NM_031530 | Rattus norvegicus chemokine (C-C motif) ligand 2 (Ccl2), mRNA. | 932.5 | **0.000** | 3208.1 | **0.000** | **3.440** | 0.373 |
| NM_013025 | Rattus norvegicus chemokine (C-C motif) ligand 3 (Ccl3), mRNA. | 28.5 | **0.005** | 48.4 | **0.002** | **1.696** | 0.673 |
| NM_017333 | Rattus norvegicus endothelin receptor type B (Ednrb), mRNA. | 62.8 | **0.002** | 68.9 | **0.002** | 1.097 | 0.746 |
| NM_031512 | Rattus norvegicus interleukin 1 beta (Il1b), mRNA. | 64.1 | **0.002** | 109.5 | **0.000** | **1.709** | 0.676 |
| NM_012713 | Rattus norvegicus protein kinase C, beta 1 (Prkcb1), mRNA. | 81.7 | **0.001** | 52.0 | **0.002** | **0.637** | 0.562 |
| NM_017059 | Rattus norvegicus Bcl2-associated X protein (Bax), mRNA. | 24.4 | **0.007** | 23.6 | **0.005** | 0.966 | 0.905 |
| NM_023970 | Rattus norvegicus transient receptor potential cation channel, subfamily V, member 4 (Trpv4), mRNA. | 65.0 | **0.002** | 27.7 | **0.004** | **0.426** | **0.000** |
