# Supplementary material for: Functional Comparison of Chronological and In Vitro Aging: Differential Role of the Cytoskeleton and Mitochondria in Mesenchymal Stromal Cells
Source: PLoS One. 2012 Dec 28;7(12):e52700. doi: 10.1371/journal.pone.0052700 (PMC3532360; doi:10.1371/journal.pone.0052700)
Supplement: Table S4 — Exclusively expressed mRNAs in long-term cultivated MSCs of passage 30 and 100. (DOC) [file pone.0052700.s007.doc]

**Table S4: Exclusively expressed mRNAs in long-term cultivated MSCs of passage 30 and 100**

| **Notch signaling pathway (p=0,021; n=3)** | |  |  |  |  |  |  |  |  |
| --- | --- | --- | --- | --- | --- | --- | --- | --- | --- |
| ACCESSION | Name | yMSCs P 30 | | aMSCs P30 | | yMSCs P100 | | aMSCs P100 | |
|  |  | AVG | P_Det_. | AVG | P_Det_. | AVG | P_Det_. | AVG | P_Det_. |
| XM_576312 | PREDICTED: Rattus norvegicus E1A binding protein p300 (Ep300), mRNA. | 29.6 | **0.007** | 24.9 | **0.007** | 25.9 | **0.007** | 26.2 | **0.006** |
| NM_133393 | Rattus norvegicus lunatic fringe gene homolog (Drosophila) (Lfng), mRNA. | 41.3 | **0.004** | 27.5 | **0.006** | 31.9 | **0.006** | 20.2 | **0.008** |
| XM_342392 | PREDICTED: Rattus norvegicus Notch gene homolog 1, (Drosophila) (Notch1), mRNA. | 47.4 | **0.004** | 49.3 | **0.002** | 23.2 | **0.008** | 61.4 | **0.004** |
| **Cell cycle (p=0,015; n=7)** | |  |  |  |  |  |  |  |  |
| ACCESSION | Name | yMSCs P 30 | | aMSCs P30 | | yMSCs P100 | | aMSCs P100 | |
|  |  | AVG | P_Det_. | AVG | P_Det_. | AVG | P_Det_. | AVG | P_Det_. |
| XM_341713 | PREDICTED: Fanconi anemia, complementation group A (predicted) (Fanca_predicted), mRNA. | 70.0 | **0.002** | 78.5 | **0.000** | 54.4 | **0.002** | 46.0 | **0.004** |
| NM_133393 | lunatic fringe gene homolog (Drosophila) (Lfng), mRNA. | 41.3 | **0.004** | 27.5 | **0.006** | 31.9 | **0.006** | 20.2 | **0.008** |
| XM_216161 | PREDICTED: MAD2 (mitotic arrest deficient, homolog)-like 1 (yeast) (predicted) (Mad2l1_predicted), mRNA. | 38.5 | **0.004** | 30.9 | **0.004** | 38.2 | **0.004** | 28.2 | **0.006** |
| XM_575211 | PREDICTED: similar to DNA repair protein RAD51 homolog 1 (LOC499870), mRNA. | 35.6 | **0.004** | 23.3 | **0.008** | 38.1 | **0.004** | 27.2 | **0.006** |
| XM_235691 | PREDICTED: extra spindle poles like 1 (S. cerevisiae) (predicted) (Espl1_predicted), mRNA. | 86.6 | **0.002** | 103.1 | **0.000** | 90.2 | **0.001** | 55.6 | **0.004** |
| NM_153470 | leucine zipper, putative tumor suppressor 1 (Lzts1), mRNA. | 37.5 | **0.004** | 28.7 | **0.006** | 40.4 | **0.002** | 59.0 | **0.004** |
| XM_575668 | PREDICTED: similar to mKIAA0159 protein (LOC362438), mRNA. | 85.8 | **0.002** | 89.0 | **0.000** | 70.7 | **0.002** | 75.2 | **0.004** |
| **Receptor (p=0,034; n=11)** | |  |  |  |  |  |  |  |  |
| ACCESSION | Name | yMSCs P 30 | | aMSCs P30 | | yMSCs P100 | | aMSCs P100 | |
|  |  | AVG | P_Det_. | AVG | P_Det_. | AVG | P_Det_. | AVG | P_Det_. |
| NM_012852 | Rattus norvegicus 5-hydroxytryptamine (serotonin) receptor 1D (Htr1d), mRNA. | 31.4 | **0.005** | 28.4 | **0.006** | 30.7 | **0.006** | 27.1 | **0.006** |
| XM_233574 | PREDICTED: Rattus norvegicus similar to Ephb2 protein (LOC313633), mRNA. | 47.2 | **0.004** | 37.7 | **0.004** | 41.8 | **0.002** | 64.7 | **0.004** |
| XM_342392 | PREDICTED: Rattus norvegicus Notch gene homolog 1, (Drosophila) (Notch1), mRNA. | 47.4 | **0.004** | 49.3 | **0.002** | 23.2 | **0.008** | 61.4 | **0.004** |
| NM_017078 | Rattus norvegicus cholinergic receptor, nicotinic, alpha polypeptide 5 (Chrna5), mRNA. | 22.5 | **0.010** | 25.4 | **0.007** | 24.2 | **0.008** | 23.8 | **0.006** |
| NM_012832 | Rattus norvegicus cholinergic receptor, nicotinic, alpha polypeptide 7 (Chrna7), mRNA. | 26.4 | **0.007** | 24.8 | **0.007** | 24.5 | **0.008** | 23.4 | **0.006** |
| XM_224604 | interleukin 17 receptor B | 22.1 | **0.010** | 23.5 | **0.008** | 21.7 | **0.008** | 22.7 | **0.007** |
| NM_001000998 | Rattus norvegicus olfactory receptor 1073 (predicted) (Olr1073_predicted), mRNA. | 32.5 | **0.005** | 32.3 | **0.004** | 27.6 | **0.007** | 27.2 | **0.006** |
| NM_033441 | Rattus norvegicus rhodopsin (Rho), mRNA. | 26.8 | **0.007** | 28.8 | **0.006** | 25.5 | **0.007** | 27.2 | **0.006** |
| XM_576311 | PREDICTED: Rattus norvegicus androgen receptor-related apoptosis-associated protein CBL27 (Cbl27), mRNA. | 30.1 | **0.007** | 27.7 | **0.006** | 24.6 | **0.008** | 33.9 | **0.005** |
| NM_001008961 | Rattus norvegicus vomeronasal 1 receptor, e7 (V1re7), mRNA. | 27.2 | **0.007** | 29.5 | **0.004** | 29.8 | **0.006** | 22.5 | **0.007** |
| XM_222983 | PREDICTED: Rattus norvegicus similar to putative pheromone receptor (Go-VN2) (LOC289295), mRNA. | 26.9 | **0.007** | 24.0 | **0.008** | 23.7 | **0.008** | 27.8 | **0.006** |
