# Supplementary material for: Functional Comparison of Chronological and In Vitro Aging: Differential Role of the Cytoskeleton and Mitochondria in Mesenchymal Stromal Cells
Source: PLoS One. 2012 Dec 28;7(12):e52700. doi: 10.1371/journal.pone.0052700 (PMC3532360; doi:10.1371/journal.pone.0052700)
Supplement: Table S5 — Genes with up-regulated expression after long-term cultivation. (DOC) [file pone.0052700.s008.doc]

**Table S5: Genes with up-regulated expression after long-term cultivation**

| **Cell Cycle (n=33 , p<0.001)** | | n=21 | p<0.001 | n=23 | p<0.001 | n=27 | p<0.001 | n=26 | p<0.001 | n=1 |  | n=8 | p<0.01 |
| --- | --- | --- | --- | --- | --- | --- | --- | --- | --- | --- | --- | --- | --- |
| ACCESSION | Name | yMSCs | | aMSCs | | yMSCs | | aMSCs | | aMSCs/yMSCs | | aMSCs/yMSCs | |
|  |  | P30/P2 | | P30/P2 | | P100/P2 | | P100/P2 | | P2 | | P100 | |
|  |  | Ratio | p-value | Ratio | p-value | Ratio | p-value | Ratio | p-value | Ratio | p-value | Ratio | p-value |
| XM_230589 | anaphase promoting complex subunit 1 (Anapc1 ) | **2.779** | 0.079 | **3.051** | **0.000** | 1.336 | **0.004** | **3.317** | **0.033** | 0.811 | 0.178 | **2.013** | 0.078 |
| XM_223496 | anaphase promoting complex subunit 4 (Anapc4 ) | 1.314 | **0.048** | 1.374 | 0.093 | 1.306 | **0.035** | **1.578** | **0.025** | 0.997 | 0.985 | 1.205 | 0.138 |
| XM_213783 | anaphase-promoting complex subunit 5 (Anapc5 ) | 1.202 | 0.190 | **1.670** | **0.008** | 1.156 | 0.280 | **1.547** | **0.015** | 0.863 | 0.306 | 1.155 | 0.135 |
| XM_215849 | budding uninhibited by benzimidazoles 1 homolog (Bub1 ) | **2.755** | **0.012** | **2.623** | **0.009** | **2.059** | **0.015** | **2.461** | **0.039** | 0.856 | 0.615 | 1.022 | 0.713 |
| XM_342494 | budding uninhibited by benzimidazoles 1 homolog, beta(Bub1b) | **4.853** | **0.008** | **5.020** | **0.001** | **4.607** | **0.000** | **5.964** | **0.008** | 0.849 | 0.511 | 1.099 | 0.661 |
| NM_019296 | cell division cycle 2 homolog A (S. pombe) (Cdc2a) | **2.823** | **0.018** | **3.668** | **0.008** | **3.121** | **0.001** | **2.751** | **0.009** | 0.712 | 0.300 | **0.628** | **0.000** |
| NM_171993 | cell division cycle 20 homolog(Cdc20) | **3.738** | **0.004** | **5.918** | **0.000** | **3.961** | **0.001** | **2.236** | **0.002** | 0.744 | 0.409 | **0.420** | **0.000** |
| XM_340896 | cell division cycle 6 homolog (Cdc6 ) | **2.848** | **0.001** | **5.193** | **0.000** | **4.303** | **0.000** | **3.392** | **0.006** | **0.669** | 0.208 | **0.528** | **0.000** |
| NM_001009470 | cyclin B2 (Ccnb2) | **2.547** | **0.028** | **4.085** | **0.000** | **2.899** | **0.004** | **3.090** | **0.001** | 0.812 | 0.569 | 0.866 | 0.257 |
| NM_171992 | cyclin D1 (Ccnd1) | 1.344 | 0.152 | 1.074 | 0.284 | **2.309** | **0.000** | 0.955 | 0.963 | **1.795** | **0.000** | 0.742 | **0.048** |
| XM_342804 | cyclin E2 (Ccne2 ) | **1.920** | **0.039** | 1.319 | 0.457 | **1.710** | **0.017** | 1.410 | 0.298 | **0.674** | 0.188 | **0.555** | 0.091 |
| NM_053593 | cyclin-dependent kinase 4 (Cdk4) | 1.340 | 0.080 | 1.269 | **0.039** | 1.466 | **0.004** | **1.507** | **0.008** | 0.807 | 0.106 | 0.829 | 0.057 |
| NM_031762 | cyclin-dependent kinase inhibitor 1B (Cdkn1b) | 0.950 | 0.830 | 0.874 | 0.550 | 1.055 | 0.829 | **1.958** | **0.042** | 0.838 | 0.425 | **1.556** | 0.198 |
| NM_131902 | cyclin-dependent kinase inhibitor 2C (p18, inhibits CDK4) (Cdkn2c) | 1.000 | 1.000 | 1.000 | 1.000 | 1.000 | 1.000 | **3.862** | **0.004** | 1.217 | 0.836 | 1.215 | 0.383 |
| XM_230765 | E2F transcription factor 1 (E2f1) | **1.839** | **0.001** | **3.692** | **0.000** | **1.631** | **0.027** | **1.572** | 0.066 | 0.723 | 0.168 | 0.697 | 0.090 |
| XM_235691 | extra spindle poles like 1 (Espl1 ) | **8.512** | **0.041** | **15.063** | **0.009** | **8.869** | **0.002** | **8.125** | **0.001** | **0.673** | 0.558 | **0.616** | **0.026** |
| XM_243390 | fizzy/cell division cycle 20 related 1 (Fzr1 ) | **1.801** | 0.052 | **3.122** | **0.003** | **1.723** | **0.022** | **2.416** | **0.036** | 0.784 | 0.432 | 1.099 | 0.471 |
| XM_216161 | MAD2 (mitotic arrest deficient, homolog)-like 1 (yeast) (Mad2l1 ) | **2.686** | 0.056 | **3.237** | **0.002** | **2.666** | **0.011** | **2.952** | **0.042** | **0.666** | 0.546 | 0.738 | 0.271 |
| XM_344048 | mini chromosome maintenance deficient 4 homolog(Mcmd4) | **1.901** | **0.027** | **1.880** | **0.014** | **1.795** | **0.001** | **1.521** | **0.049** | 1.045 | 0.804 | 0.885 | 0.592 |
| XM_344135 | mini chromosome maintenance deficient 6(Mcmd6) | **5.479** | **0.003** | **6.047** | **0.000** | **5.506** | **0.000** | **5.498** | **0.022** | 0.925 | 0.710 | 0.924 | 0.757 |
| XM_232168 | minichromosome maintenance deficient 2 mitotin (Mcm2 ) | **3.210** | **0.030** | **3.004** | **0.003** | **2.648** | **0.001** | **3.431** | **0.025** | 0.741 | **0.034** | 0.960 | 0.898 |
| XM_236988 | minichromosome maintenance deficient 3 (Mcm3 ) | **4.505** | **0.001** | **5.504** | **0.000** | **4.987** | **0.000** | **3.208** | **0.011** | 0.863 | 0.592 | **0.555** | **0.000** |
| XM_226316 | minichromosome maintenance deficient 5, cell division cycle 46 (Mcm5 ) | **4.934** | **0.005** | **3.694** | 0.097 | **8.628** | **0.000** | **8.738** | **0.017** | 0.758 | 0.428 | 0.768 | 0.226 |
| NM_001004203 | minichromosome maintenance deficient 7(Mcm7) | **3.711** | **0.000** | **4.793** | **0.000** | **4.549** | **0.000** | **3.606** | **0.010** | 0.745 | 0.157 | **0.591** | **0.001** |
| NM_177931 | origin recognition complex, subunit 1-like (S.cereviaiae) (Orc1l) | **3.751** | **0.018** | **6.090** | **0.000** | **5.335** | **0.001** | **2.860** | **0.040** | 0.709 | 0.359 | **0.380** | **0.000** |
| NM_017100 | polo-like kinase 1 (Plk1) | **4.670** | **0.000** | **8.633** | **0.000** | **4.993** | **0.001** | **4.371** | **0.006** | 0.813 | 0.546 | 0.712 | **0.036** |
| NM_053677 | protein kinase Chk2 (Chek2) | **1.827** | **0.001** | **2.532** | **0.004** | **2.095** | **0.000** | **3.128** | **0.004** | 0.831 | 0.342 | 1.241 | 0.195 |
| XM_346005 | similar to ataxia telangiectasia and Rad3 related protein (LOC367198) | **1.626** | 0.132 | 1.399 | 0.061 | **2.071** | **0.002** | **1.568** | **0.034** | 1.039 | 0.822 | 0.778 | **0.018** |
| XM_231388 | similar to DBF4-related protein (LOC312046) | **3.078** | **0.001** | **2.064** | **0.014** | **3.409** | **0.002** | **2.310** | **0.013** | 1.098 | 0.702 | 0.744 | 0.051 |
| XM_213222 | similar to membrane-associated tyrosine-and threonine-specific cdc2-inhibitory kinase | **2.913** | **0.006** | **3.890** | **0.022** | **3.741** | **0.002** | **2.860** | **0.002** | **0.644** | 0.268 | **0.493** | **0.000** |
| XM_226817 | similar to S-phase kinase-associated protein 2 ( (Cyclin A/CDK2-associated protein p45) | **1.820** | 0.060 | 0.884 | 0.826 | **1.841** | **0.000** | **1.718** | 0.126 | 0.765 | 0.276 | 0.714 | 0.287 |
| NM_031683 | structural maintenance of chromosomes 1 like 1(Smc1l1) | **2.284** | **0.003** | **1.962** | **0.003** | **2.243** | **0.001** | **2.506** | **0.017** | 1.050 | 0.713 | 1.173 | 0.429 |
| NM_030989 | tumor protein p53 (Tp53) | 1.441 | **0.041** | **1.729** | **0.022** | **2.285** | **0.001** | **1.877** | **0.019** | 0.980 | 0.951 | 0.805 | 0.152 |
| **p53 signaling (n=21 , p<0.001)** | | n=9 | p<0.001 | n=10 | p<0.001 | n=10 | p<0.001 | n=18 | p<0.001 | n=4 | p<0.001 | n=6 | p<0.001 |
| ACCESSION | Name | yMSCs | | aMSCs | | yMSCs | | aMSCs | | aMSCs/yMSCs | | aMSCs/yMSCs | |
|  |  | P30/P2 | | P30/P2 | | P100/P2 | | P100/P2 | | P2 | | P100 | |
|  |  | Ratio | p-value | Ratio | p-value | Ratio | p-value | Ratio | p-value | Ratio | p-value | Ratio | p-value |
| NM_053677 | protein kinase Chk2 (Chek2), mRNA. | **1.827** | **0.001** | **2.532** | **0.004** | **2.095** | **0.000** | **3.128** | **0.004** | 0.831 | 0.342 | 1.241 | 0.195 |
| NM_012680 | tuberous sclerosis 2 (Tsc2), mRNA. | **1.940** | 0.129 | **1.656** | **0.027** | 1.472 | **0.004** | **2.007** | **0.047** | 1.000 | 0.999 | 1.363 | 0.292 |
| XM_346005 | similar to ataxia telangiectasia and Rad3 related protein (LOC367198), mRNA. | **1.626** | 0.132 | 1.399 | 0.061 | **2.071** | **0.002** | **1.568** | **0.034** | 1.039 | 0.822 | 0.778 | **0.018** |
| NM_171992 | cyclin D1 (Ccnd1), mRNA. | 1.344 | 0.152 | 1.074 | 0.284 | **2.309** | **0.000** | 0.955 | 0.963 | **1.795** | **0.000** | 0.742 | **0.048** |
| NM_019296 | cell division cycle 2 homolog A (S. pombe) (Cdc2a), mRNA. | **2.823** | **0.018** | **3.668** | **0.008** | **3.121** | **0.001** | **2.751** | **0.009** | 0.712 | 0.300 | **0.628** | **0.000** |
| XM_342804 | cyclin E2 (Ccne2 ), mRNA. | **1.920** | **0.039** | 1.319 | 0.457 | **1.710** | **0.017** | 1.410 | 0.298 | **0.674** | 0.188 | **0.555** | **0.036** |
| NM_053593 | cyclin-dependent kinase 4 (Cdk4), mRNA. | 1.340 | 0.080 | 1.269 | **0.039** | 1.466 | **0.004** | **1.507** | **0.008** | 0.807 | 0.106 | 0.829 | 0.135 |
| NM_001009470 | cyclin B2 (Ccnb2), mRNA. | **2.547** | **0.028** | **4.085** | **0.000** | **2.899** | **0.004** | **3.090** | **0.001** | 0.812 | 0.569 | 0.866 | 0.257 |
| NM_030989 | tumor protein p53 (Tp53), mRNA. | 1.441 | **0.041** | **1.729** | **0.022** | **2.285** | **0.001** | **1.877** | **0.019** | 0.980 | 0.951 | 0.805 | 0.152 |
| XM_217016 | similar to B99 protein (LOC300126), mRNA. | **2.151** | **0.005** | **2.141** | **0.007** | **2.641** | **0.001** | **1.721** | **0.042** | 0.840 | 0.387 | **0.547** | **0.000** |
| NM_001007754 | Ras association (RalGDS/AF-6) domain family 1 (Rassf1), transcript variant 2, mRNA. | **2.009** | **0.004** | 1.169 | 0.316 | **1.625** | **0.016** | 0.768 | 0.091 | **1.575** | **0.004** | 0.744 | **0.037** |
| NM_031762 | cyclin-dependent kinase inhibitor 1B (p27, Cdkn1b), mRNA. | 0.950 | 0.830 | 0.874 | 0.550 | 1.055 | 0.829 | **1.958** | **0.042** | 0.838 | 0.425 | **1.556** | 0.176 |
| NM_131902 | cyclin-dependent kinase inhibitor 2C (p18, inhibits CDK4, Cdkn2c), mRNA. | **n.d** |  | **n.d** |  | **n.d** |  | **3.862** | **0.004** | 1.217 | 0.836 | 1.215 | 0.584 |
| NM_080782 | cyclin-dependent kinase inhibitor 1A (p21, Cdkn1a), mRNA. | **0.278** | **0.004** | **0.423** | **0.012** | 0.767 | 0.156 | **0.321** | **0.000** | **0.641** | **0.005** | **0.268** | **0.000** |
| NM_012922 | caspase 3, apoptosis related cysteine protease (Casp3), mRNA. | 1.246 | 0.070 | 1.357 | **0.026** | 1.256 | 0.138 | **1.631** | **0.009** | 0.963 | 0.783 | 1.250 | 0.190 |
| NM_001009470 | cyclin B2 (Ccnb2), mRNA. | **2.547** | **0.028** | **4.085** | **0.000** | **2.899** | **0.004** | **3.090** | **0.001** | 0.812 | 0.569 | 0.866 | 0.257 |
| NM_053593 | cyclin-dependent kinase 4 (Cdk4), mRNA. | 1.340 | 0.080 | 1.269 | **0.039** | 1.466 | **0.004** | **1.507** | **0.008** | 0.807 | 0.106 | 0.829 | 0.135 |
| XM_235825 | sestrin 3 (Sesn3 ), mRNA. | 1.033 | 0.726 | **2.785** | **0.030** | 0.815 | 0.201 | **6.831** | **0.001** | 0.822 | 0.394 | **6.889** | **0.000** |
| NM_001006989 | scotin (MGC94600), mRNA. | **0.644** | **0.003** | 0.956 | 0.723 | 0.948 | 0.574 | **1.699** | **0.000** | **0.613** | **0.030** | 1.099 | 0.715 |
| NM_023979 | apoptotic peptidase activating factor 1 (Apaf1), mRNA. | **1.846** | 0.099 | **1.715** | **0.013** | 1.365 | **0.019** | **1.770** | **0.015** | 0.862 | 0.364 | 1.118 | 0.506 |
| XM_215423 | sestrin 1 (Sesn1 ), mRNA. | 0.859 | 0.688 | 1.391 | 0.157 | 0.711 | **0.042** | **3.390** | **0.007** | 0.730 | **0.023** | **3.484** | **0.000** |
| **DNA replication (n=14 , p<0.001)** | | n=12 | p<0.001 | n=12 | p<0.001 | n=13 | p<0.001 | n=14 | p<0.001 | n=2 |  | n=6 | p<0.001 |
| ACCESSION | Name | yMSCs | | aMSCs | | yMSCs | | aMSCs | | aMSCs/yMSCs | | aMSCs/yMSCs | |
|  |  | P30/P2 | | P30/P2 | | P100/P2 | | P100/P2 | | P2 | | P100 | |
|  |  | Ratio | p-value | Ratio | p-value | Ratio | p-value | Ratio | p-value | Ratio | p-value | Ratio | p-value |
| NM_053430 | flap structure-specific endonuclease 1 (Fen1) | **2.342** | **0.003** | **2.594** | **0.009** | **3.004** | **0.000** | **1.685** | **0.003** | 0.910 | 0.655 | **0.511** | **0.000** |
| XM_344048 | mini chromosome maintenance deficient 4 homolog (S. cerevisiae) (Mcmd4) | **1.901** | **0.027** | **1.880** | **0.014** | **1.795** | **0.001** | **1.521** | **0.049** | 1.045 | 0.804 | 0.885 | 0.464 |
| XM_344135 | mini chromosome maintenance deficient 6 (S. cerevisiae) (Mcmd6) | **5.479** | **0.003** | **6.047** | **0.000** | **5.506** | **0.000** | **5.498** | **0.022** | 0.925 | 0.710 | 0.924 | 0.757 |
| XM_232168 | minichromosome maintenance deficient 2 mitotin (S. cerevisiae) (Mcm2 ) | **3.210** | **0.030** | **3.004** | **0.003** | **2.648** | **0.001** | **3.431** | **0.025** | 0.741 | **0.034** | 0.960 | 0.898 |
| XM_242396 | polymerase (DNA directed), alpha 1 (Pola1) | **3.522** | **0.020** | 1.221 | 0.509 | **3.413** | **0.002** | **2.624** | **0.021** | 1.107 | 0.589 | 0.851 | 0.437 |
| NM_053480 | polymerase (DNA directed), alpha 2 (Pola2) | **2.599** | **0.000** | **2.041** | **0.000** | **5.298** | **0.000** | **2.956** | **0.014** | **1.713** | **0.000** | 0.956 | 0.824 |
| NM_021662 | polymerase (DNA directed), delta 1, catalytic subunit (Pold1) | **5.381** | **0.020** | **5.609** | **0.003** | **4.417** | **0.001** | **6.458** | **0.017** | **0.591** | **0.029** | 0.864 | 0.557 |
| XM_216727 | polymerase (DNA directed), epsilon 2 (p59 subunit) (Pole2 ) | **4.747** | **0.000** | **3.707** | **0.010** | **6.430** | **0.000** | **5.631** | **0.001** | 0.695 | 0.122 | **0.608** | **0.000** |
| NM_001009629 | replication factor C (activator 1) 3 (Rfc3) | **1.987** | 0.088 | **2.679** | **0.002** | **2.151** | **0.006** | **2.141** | **0.003** | 0.786 | 0.354 | 0.782 | **0.050** |
| XM_213598 | replication factor C (activator 1) 4 (Rfc4 ) | 1.240 | 0.328 | **1.646** | **0.013** | 1.298 | 0.091 | **1.598** | **0.023** | 0.790 | 0.121 | 0.973 | 0.841 |
| XM_222214 | replication factor C (activator 1) 5 (Rfc5 ) | **1.939** | **0.012** | **2.409** | **0.005** | **2.149** | **0.002** | **1.744** | **0.001** | 0.719 | 0.085 | **0.584** | **0.000** |
| XM_226316 | minichromosome maintenance deficient 5, cell division cycle 46 (S. cerevisiae) (Mcm5 ) | **4.934** | **0.005** | **3.694** | 0.097 | **8.628** | **0.000** | **8.738** | **0.017** | 0.758 | 0.428 | 0.768 | 0.226 |
| NM_001004203 | minichromosome maintenance deficient 7 (S. cerevisiae) (Mcm7) | **3.711** | **0.000** | **4.793** | **0.000** | **4.549** | **0.000** | **3.606** | **0.010** | 0.745 | 0.157 | **0.591** | **0.001** |
| XM_222255 | polymerase (DNA directed), epsilon (Pole) | **6.335** | **0.026** | **5.912** | **0.001** | **5.642** | **0.000** | **4.131** | **0.009** | 0.858 | 0.602 | **0.628** | **0.007** |
| XM_236988 | minichromosome maintenance deficient 3 (S. cerevisiae) (Mcm3 ) | **4.505** | **0.001** | **5.504** | **0.000** | **4.987** | **0.000** | **3.208** | **0.011** | 0.863 | 0.592 | **0.555** | **0.000** |
| NM_001008768 | DNA primase, p49 subunit (Prim1) | **2.289** | **0.003** | **2.330** | **0.003** | **2.710** | **0.001** | **2.323** | **0.001** | 0.830 | 0.182 | 0.712 | **0.008** |
| **MAPK (n=17 , p<0.001)** | | n=8 | p<0.001 | n=10 | p<0.001 | n=7 | p<0.001 | n=17 | p<0.001 | n=1 |  | n=9 | p<0.001 |
| ACCESSION | Name | yMSCs | | aMSCs | | yMSCs | | aMSCs | | aMSCs/yMSCs | | aMSCs/yMSCs | |
|  |  | P30/P2 | | P30/P2 | | P100/P2 | | P100/P2 | | P2 | | P100 | |
|  |  | Ratio | p-value | Ratio | p-value | Ratio | p-value | Ratio | p-value | Ratio | p-value | Ratio | p-value |
| NM_012828 | calcium channel, voltage-dependent, beta 3 subunit (Cacnb3) | **2.284** | **0.005** | **2.309** | **0.001** | **1.884** | **0.001** | **3.440** | **0.006** | 0.683 | **0.006** | 1.247 | 0.197 |
| NM_012517 | calcium channel, voltage-dependent, L type, alpha 1C subunit (Cacna1c) | **2.161** | **0.046** | **0.661** | 0.083 | 1.493 | **0.001** | **2.815** | **0.000** | 1.016 | 0.927 | **1.916** | **0.001** |
| XM_579518 | calcium channel, voltage-dependent, T type, alpha 1G subunit (Cacna1g) | **1.763** | 0.249 | 1.000 | 1.000 | **0.336** | 0.063 | **6.728** | **0.032** | **0.460** | 0.162 | **9.209** | **0.005** |
| NM_012922 | caspase 3, apoptosis related cysteine protease (Casp3) | 1.246 | 0.070 | 1.357 | **0.026** | 1.256 | 0.138 | **1.631** | **0.009** | 0.963 | 0.783 | 1.250 | 0.105 |
| XM_223030 | dual specificity phosphatase 10 (Dusp10 ) | 1.000 | 1.000 | **2.393** | 0.270 | 1.000 | 1.000 | **3.335** | **0.004** | 1.113 | 0.826 | **7.312** | **0.000** |
| XM_238551 | dual specificity phosphatase 7 (Dusp7) | 1.243 | 0.200 | **1.733** | 0.124 | 1.000 | 1.000 | **2.135** | **0.015** | 0.815 | 0.589 | 1.463 | 0.249 |
| NM_031507 | epidermal growth factor receptor (Egfr) | **1.725** | 0.319 | 1.211 | 0.585 | 1.000 | 1.000 | **1.976** | **0.042** | 1.287 | 0.573 | **3.528** | **0.003** |
| NM_022182 | fibroblast growth factor 7 (Fgf7) | **0.146** | **0.007** | **0.214** | **0.003** | **0.252** | **0.011** | **1.788** | **0.004** | **0.311** | **0.000** | **2.207** | **0.001** |
| XM_579712 | MAP kinase-activated protein kinase 2 (Mapkapk2) | 1.441 | 0.063 | **2.670** | **0.017** | 0.850 | 0.052 | **1.581** | **0.050** | 0.759 | **0.043** | 1.412 | **0.045** |
| XM_576180 | MAP kinase-interacting serine/threonine kinase 2 (predicted) | 1.288 | 0.119 | 1.334 | **0.009** | 1.370 | **0.003** | **2.444** | **0.005** | 0.943 | 0.654 | **1.683** | **0.001** |
| XM_239239 | mitogen activated protein kinase kinase 3 (Map2k3 ) | 1.249 | **0.010** | 1.121 | 0.244 | 1.247 | **0.030** | **1.690** | **0.001** | 1.042 | 0.753 | 1.413 | **0.024** |
| XM_574265 | mitogen-activated protein kinase kinase kinase 7 interacting protein 2 | 1.253 | 0.062 | 1.260 | 0.068 | 1.059 | 0.281 | 1.462 | **0.042** | 0.979 | 0.870 | 1.351 | 0.068 |
| XM_343006 | mitogen-activated protein kinase kinase kinase kinase 3 (Map4k3) | **2.514** | **0.001** | 0.850 | 0.237 | **1.543** | **0.000** | **2.213** | **0.004** | 1.290 | 0.054 | **1.850** | **0.000** |
| NM_012981 | muscle and microspikes RAS (Mras) | **1.662** | 0.165 | **2.690** | **0.005** | 1.320 | **0.033** | **3.156** | **0.031** | **0.589** | **0.012** | 1.408 | 0.189 |
| NM_012603 | myelocytomatosis viral oncogene homolog (avian) (Myc) | 1.345 | **0.016** | **2.199** | **0.008** | 1.410 | **0.007** | **1.515** | **0.040** | 0.757 | **0.042** | 0.813 | 0.213 |
| NM_133551 | phospholipase A2, group IVA (cytosolic, calcium-dependent) (Pla2g4a) | **1.597** | **0.034** | 1.177 | 0.224 | 1.187 | 0.274 | **1.705** | **0.028** | 0.882 | 0.435 | 1.267 | 0.126 |
| XM_341661 | protein kinase, cAMP-dependent, catalytic, alpha (Prkaca) | 1.138 | 0.112 | 1.223 | 0.474 | 0.980 | 0.956 | **1.545** | **0.047** | 0.833 | 0.439 | 1.313 | 0.066 |
| NM_017041 | protein phosphatase 3, catalytic subunit, alpha isoform (Ppp3ca) | 1.287 | 0.062 | 1.181 | 0.497 | 1.386 | **0.034** | **2.317** | **0.001** | 0.930 | 0.585 | **1.555** | **0.007** |
| XM_233873 | RAS, guanyl releasing protein 3 (Rasgrp3 ) | **0.260** | **0.006** | **0.204** | **0.001** | **0.371** | **0.014** | **1.547** | **0.031** | 1.294 | 0.153 | **5.399** | **0.001** |
| XM_342004 | ribosomal protein S6 kinase, polypeptide 4 (predicted) | 1.326 | 0.254 | **1.999** | **0.026** | 1.203 | 0.422 | **2.101** | **0.026** | 0.759 | 0.221 | 1.326 | 0.252 |
| XM_234468 | ribosomal protein S6 kinase, polypeptide 5 (predicted) | 1.000 | 1.000 | 1.000 | 1.000 | 1.000 | 1.000 | **3.815** | **0.005** | **0.416** | 0.172 | 1.375 | 0.451 |
| XM_220874 | similar to Stathmin (Phosphoprotein p19) (pp19) | **3.204** | **0.007** | **4.316** | **0.003** | **3.287** | **0.021** | **2.754** | **0.022** | 0.896 | 0.608 | 0.751 | 0.261 |
| NM_017166 | stathmin 1 (Stmn1) | **1.992** | **0.028** | **2.296** | **0.014** | **2.301** | **0.009** | **1.705** | **0.040** | 0.870 | 0.615 | **0.644** | **0.001** |
| NM_030989 | tumor protein p53 (Tp53) | 1.441 | **0.041** | **1.729** | **0.022** | **2.285** | **0.001** | **1.877** | **0.019** | 0.980 | 0.951 | 0.805 | 0.152 |
| **Insulin signaling &**  **phosphatidylinositol signaling system (n=19 , p<0.001)** | | n=4 | p<0.02 | n=9 | p<0.001 | n=2 |  | n=19 | p<0.001 | n=2 |  | n=10 | p<0.001 |
| ACCESSION | Name | yMSCs | | aMSCs | | yMSCs | | aMSCs | | aMSCs/yMSCs | | aMSCs/yMSCs | |
|  |  | P30/P2 | | P30/P2 | | P100/P2 | | P100/P2 | | P2 | | P100 | |
|  |  | Ratio | p-value | Ratio | p-value | Ratio | p-value | Ratio | p-value | Ratio | p-value | Ratio | p-value |
| NM_031081 | 3-phosphoinositide dependent protein kinase-1 (Pdpk1) | **1.794** | **0.005** | **1.877** | **0.008** | 1.422 | **0.008** | **1.555** | **0.010** | 1.083 | 0.589 | 1.184 | 0.243 |
| NM_031143 | diacylglycerol kinase zeta (Dgkz) | **1.951** | 0.123 | **5.247** | **0.021** | **1.722** | 0.052 | **5.063** | **0.010** | **0.550** | 0.083 | **1.618** | 0.072 |
| NM_022701 | flotillin 1 (Flot1) | **1.679** | **0.017** | 1.382 | 0.194 | 1.322 | **0.043** | **1.707** | **0.004** | 1.314 | 0.061 | **1.696** | **0.001** |
| XM_579364 | hypothetical gene supported by NM_013005 (LOC497750) | 1.227 | 0.269 | 1.084 | 0.351 | 1.301 | 0.127 | **2.055** | **0.002** | 0.796 | 0.368 | 1.257 | 0.298 |
| NM_172224 | inositol (myo)-1(or 4)-monophosphatase 2 (Impa2) | 1.249 | 0.454 | **2.872** | **0.001** | 1.360 | 0.136 | **6.011** | **0.002** | **0.615** | **0.045** | **2.718** | **0.000** |
| NM_022944 | inositol polyphosphate phosphatase-like 1 (Inppl1) | **1.749** | **0.035** | **3.008** | **0.039** | 1.351 | **0.034** | **1.551** | **0.027** | 0.978 | 0.871 | 1.123 | 0.541 |
| XM_576180 | MAP kinase-interacting serine/threonine kinase 2 (Mknk2 ) | 1.288 | 0.119 | 1.334 | **0.009** | 1.370 | **0.003** | **2.444** | **0.005** | 0.943 | 0.654 | **1.683** | **0.001** |
| NM_022185 | phosphatidylinositol 3-kinase, regulatory subunit, polypeptide 2 (Pik3r2) | 1.015 | 0.975 | 0.960 | 0.991 | 0.801 | 0.338 | **2.642** | **0.038** | **0.644** | 0.282 | **2.125** | **0.008** |
| NM_031083 | phosphatidylinositol 4-kinase, catalytic, beta polypeptide (Pik4cb) | 1.365 | 0.201 | **1.645** | **0.029** | 1.173 | 0.328 | **2.609** | **0.002** | 0.847 | 0.300 | **1.884** | **0.000** |
| NM_013190 | phosphofructokinase, liver, B-type (Pfkl) | **1.640** | 0.118 | **6.010** | **0.030** | **1.761** | **0.024** | **5.587** | **0.016** | **0.423** | **0.025** | 1.342 | 0.262 |
| NM_022958 | phosphoinositide-3-kinase, class 3 (Pik3c3) | 1.149 | 0.464 | 1.174 | 0.246 | 0.927 | 0.391 | **1.891** | **0.040** | 1.014 | 0.922 | **2.068** | **0.009** |
| NM_017035 | phospholipase C, delta 1 (Plcd1) | 1.214 | 0.139 | 1.409 | **0.043** | 0.985 | 0.676 | **1.882** | **0.023** | 0.844 | 0.203 | **1.613** | **0.017** |
| NM_053758 | phospholipase C, epsilon 1 (Plce1) | 1.000 | 1.000 | 1.000 | 1.000 | 1.000 | 1.000 | **6.210** | **0.001** | 0.812 | 0.736 | **3.008** | **0.001** |
| XM_341661 | protein kinase, cAMP-dependent, catalytic, alpha (Prkaca) | 1.138 | 0.112 | 1.223 | 0.474 | 0.980 | 0.956 | **1.545** | **0.047** | 0.833 | 0.439 | 1.313 | 0.066 |
| XM_576396 | similar to CBL E3 ubiquitin protein ligase (Signal transduction protein CBL) (Proto-oncogene c-CBL) (Casitas B-lineage lymphoma proto-oncogene) (LOC500985) | **3.401** | **0.016** | **3.034** | **0.001** | **2.238** | **0.007** | **2.875** | **0.046** | 0.828 | 0.569 | 1.064 | 0.835 |
| XM_341498 | src homology 2 domain-containing transforming protein C3 (Shc3) | 1.000 | 1.000 | 1.000 | 1.000 | 1.000 | 1.000 | **4.905** | **0.001** | 0.747 | 0.723 | **4.356** | **0.004** |
| XM_213329 | sterol regulatory element binding factor 1 (Srebf1) | **0.617** | 0.391 | **1.754** | **0.021** | **0.521** | 0.251 | **2.176** | **0.043** | **0.507** | 0.245 | **2.119** | **0.015** |
| NM_145879 | suppressor of cytokine signaling 1 (Socs1) | 1.000 | 1.000 | 1.000 | 1.000 | 1.000 | 1.000 | **5.611** | **0.015** | 1.000 | 0.821 | **2.764** | 0.053 |
| NM_012680 | tuberous sclerosis 2 (Tsc2) | **1.940** | 0.129 | **1.656** | **0.027** | 1.472 | **0.004** | **2.007** | **0.047** | 1.000 | 0.999 | 1.363 | 0.292 |
