# Supplementary material for: Functional Comparison of Chronological and In Vitro Aging: Differential Role of the Cytoskeleton and Mitochondria in Mesenchymal Stromal Cells
Source: PLoS One. 2012 Dec 28;7(12):e52700. doi: 10.1371/journal.pone.0052700 (PMC3532360; doi:10.1371/journal.pone.0052700)
Supplement: Table S6 — Genes with down-regulated expression after long-term cultivation. (DOC) [file pone.0052700.s009.doc]

**Table S6: Genes with down-regulated expression after long-term cultivation**

| **Focal adhesion (45, p<0.01)** | | n=22 | p<0.001 | n=26 | p<0.001 | n=38 | p<0.001 | n=31 | p<0.001 | n=9 | p<0.001 | n=11 | p<0.001 |
| --- | --- | --- | --- | --- | --- | --- | --- | --- | --- | --- | --- | --- | --- |
| ACCESSION | Name | yMSCs | | aMSCs | | yMSCs | | aMSCs | | aMSCs/yMSCs | | aMSCs/yMSCs | |
|  |  | P30/P2 | | P30/P2 | | P100/P2 | | P100/P2 | | P2 | | P100 | |
|  |  | Ratio | p-value | Ratio | p-value | Ratio | p-value | Ratio | p-value | Ratio | p-value | Ratio | p-value |
| NM_053653 | vascular endothelial growth factor C (Vegfc) | **0.469** | **0.009** | **0.366** | **0.009** | **0.186** | **0.002** | **0.068** | **0.001** | 0.996 | 0.979 | **0.365** | **0.000** |
| XM_214778 | thrombospondin 2 (Thbs2 ) | **0.112** | **0.001** | **0.021** | **0.004** | **0.004** | **0.001** | **0.399** | **0.029** | **0.516** | **0.001** | **50.789** | **0.000** |
| NM_017347 | mitogen activated protein kinase 3 (Mapk3) | **0.651** | **0.007** | 0.802 | 0.646 | **0.528** | **0.002** | 1.176 | 0.110 | 1.105 | 0.446 | **2.461** | **0.000** |
| NM_031836 | vascular endothelial growth factor A (Vegfa) | **0.458** | **0.007** | **0.374** | **0.015** | **0.447** | **0.006** | **0.259** | **0.000** | 0.947 | 0.697 | **0.548** | **0.000** |
| NM_019155 | caveolin 3 (Cav3) | 1.491 | 0.115 | **0.159** | **0.020** | **0.295** | **0.001** | **0.675** | 0.340 | **1.606** | 0.116 | **3.668** | **0.000** |
| XM_222794 | tenascin N (Tnn ) | **0.075** | **0.000** | **0.155** | **0.000** | **0.053** | **0.000** | **1.827** | **0.005** | **0.337** | **0.000** | **11.645** | **0.000** |
| NM_012881 | secreted phosphoprotein 1 (Spp1) | **0.244** | **0.001** | **0.484** | **0.002** | **0.073** | **0.000** | **0.269** | **0.000** | 0.865 | 0.267 | **3.190** | **0.001** |
| NM_021760 | collagen, type V, alpha 3 (Col5a3) | **0.505** | 0.237 | **2.618** | 0.148 | **0.236** | **0.019** | 0.846 | 0.621 | 0.801 | 0.717 | **2.876** | **0.012** |
| NM_012801 | platelet derived growth factor, alpha (Pdgfa) | 0.684 | 0.058 | 0.874 | 0.333 | **0.620** | **0.008** | **0.562** | **0.008** | 0.811 | 0.112 | 0.734 | **0.016** |
| XM_234523 | ELK1, member of ETS oncogene family (Elk1) | **0.430** | **0.039** | **0.152** | **0.002** | **0.338** | **0.004** | 0.724 | **0.035** | 0.916 | 0.606 | **1.965** | **0.019** |
| XM_215984 | integrin, alpha 6 (Itga6) | **1.759** | 0.186 | **0.600** | 0.342 | 0.851 | 0.436 | **0.248** | **0.001** | **1.571** | 0.105 | **0.458** | **0.021** |
| XM_579510 | hypothetical gene supported by NM_031525 (LOC497724) | 1.008 | 0.804 | 0.911 | 0.633 | **0.340** | **0.031** | **0.552** | **0.026** | 1.094 | 0.680 | **1.778** | **0.042** |
| XM_234904 | src homology 2 domain-containing transforming protein C2 (Shc2 ) | 0.828 | 0.632 | 0.858 | 0.490 | **0.206** | **0.004** | **0.411** | **0.026** | 0.939 | 0.809 | **1.871** | 0.068 |
| NM_053595 | placental growth factor (Pgf) | **0.282** | **0.029** | 0.876 | 0.566 | **0.425** | 0.052 | 0.840 | 0.583 | **0.310** | **0.001** | **0.612** | 0.073 |
| XM_230950 | integrin, alpha V (vitronectin receptor, alpha polypeptide, antigen CD51) (Itgav ) | 0.992 | 0.709 | **0.506** | **0.002** | 0.847 | 0.209 | **0.331** | **0.001** | **1.723** | **0.000** | **0.673** | 0.077 |
| XM_237444 | FERM, RhoGEF and pleckstrin domain protein 2 (Farp2 ) | 0.752 | 0.367 | **0.602** | **0.018** | **0.422** | **0.005** | 0.887 | 0.763 | 0.844 | 0.373 | **1.774** | 0.180 |
| NM_053357 | catenin (cadherin associated protein), beta 1, 88kDa (Ctnnb1) | 0.923 | 0.931 | **0.524** | **0.026** | **0.622** | 0.077 | 0.897 | 0.879 | 0.950 | 0.773 | 1.370 | 0.188 |
| NM_017093 | thymoma viral proto-oncogene 2 (Akt2) | **0.664** | **0.040** | **0.368** | 0.147 | **0.583** | **0.004** | 0.861 | 0.596 | 1.023 | 0.901 | **1.509** | 0.189 |
| NM_019143 | fibronectin 1 (Fn1) | 0.763 | 0.056 | **0.455** | **0.002** | **0.653** | **0.000** | **0.507** | **0.003** | 1.103 | 0.448 | 0.856 | 0.218 |
| XM_216688 | Rho GTPase activating protein 5 (Arhgap5 ) | **1.721** | 0.077 | **0.305** | **0.014** | 0.713 | 0.114 | **0.566** | **0.042** | **1.784** | **0.000** | 1.418 | 0.224 |
| XM_579522 | actinin alpha 4 (Actn4) | 0.992 | 0.721 | 0.797 | 0.472 | **0.598** | **0.020** | **0.543** | **0.015** | 1.263 | 0.080 | 1.147 | 0.340 |
| NM_134346 | RAS related protein 1b (Rap1b) | **0.557** | **0.000** | **0.575** | **0.001** | **0.475** | **0.000** | **0.472** | **0.000** | 1.140 | 0.312 | 1.133 | 0.341 |
| NM_053306 | p21 (CDKN1A)-activated kinase 2 (Pak2) | **0.495** | 0.077 | **0.251** | **0.012** | **0.430** | **0.045** | **0.503** | **0.028** | 1.041 | 0.849 | 1.217 | 0.408 |
| XM_230033 | integrin alpha 4 (Itga4) | 1.259 | 0.229 | **1.792** | 0.100 | **0.126** | **0.000** | **0.195** | **0.011** | 0.840 | 0.439 | 1.302 | 0.432 |
| NM_022939 | syntaxin 12 (Stx12) | **0.640** | **0.013** | **0.367** | **0.022** | **0.605** | **0.004** | **0.599** | **0.006** | 0.955 | 0.734 | 0.946 | 0.453 |
| NM_031098 | Rho-associated coiled-coil forming kinase 1 (Rock1) | **0.504** | 0.133 | **0.162** | **0.003** | **0.366** | **0.050** | **0.395** | **0.013** | 1.305 | 0.205 | 1.407 | 0.476 |
| NM_057132 | ras homolog gene family, member A (Rhoa) | **0.161** | **0.001** | **0.573** | **0.040** | **0.260** | **0.001** | **0.250** | **0.001** | 1.162 | 0.248 | 1.117 | 0.589 |
| NM_023987 | baculoviral IAP repeat-containing 3 (Birc3) | **0.294** | **0.006** | **0.333** | 0.105 | **0.348** | **0.006** | **0.360** | 0.107 | 1.410 | 0.521 | 1.459 | 0.591 |
| XM_342325 | procollagen, type XI, alpha 1 (Col11a1) | **0.013** | **0.000** | **0.079** | **0.000** | **0.002** | **0.000** | **0.004** | **0.000** | **0.486** | **0.000** | 0.857 | 0.714 |
| XM_215905 | myosin, light polypeptide 9, regulatory | **0.302** | **0.023** | **0.447** | **0.041** | **0.117** | **0.010** | **0.131** | **0.009** | 0.946 | 0.835 | 1.065 | 0.716 |
| XM_343975 | protein kinase C, alpha (Prkca) | 0.935 | 0.703 | 0.729 | **0.021** | 0.819 | 0.220 | **0.546** | **0.011** | 1.384 | **0.041** | 0.923 | 0.716 |
| NM_080394 | reelin (Reln) | **0.121** | **0.020** | **0.210** | 0.080 | **0.097** | **0.014** | **0.044** | **0.042** | **1.887** | 0.176 | 0.857 | 0.735 |
| XM_236320 | integrin, alpha 11 (Itga11 ) | 0.830 | 0.582 | 0.837 | 0.113 | **0.322** | **0.000** | **0.415** | **0.001** | 0.821 | 0.135 | 1.059 | 0.759 |
| XM_344634 | similar to integrin alpha 8 (LOC364786) | **0.436** | 0.143 | **0.196** | **0.018** | **0.359** | **0.034** | **0.168** | **0.016** | **1.831** | 0.125 | 0.857 | 0.767 |
| XM_214499 | actinin alpha 2 (Actn2 ) | **0.153** | **0.018** | 1.000 | 1.000 | **0.153** | **0.017** | 1.000 | 1.000 | **0.110** | **0.001** | 0.857 | 0.774 |
| NM_053842 | mitogen activated protein kinase 1 (Mapk1) | 0.710 | **0.006** | **0.624** | **0.001** | **0.628** | **0.002** | **0.558** | **0.001** | 1.166 | 0.241 | 1.036 | 0.782 |
| NM_012713 | protein kinase C, beta 1 (Prkcb1) | **0.086** | **0.039** | **0.135** | 0.174 | **0.086** | **0.044** | **0.115** | 0.176 | **0.637** | 0.562 | 0.857 | 0.788 |
| NM_001008384 | RAS-related C3 botulinum substrate 2 (Rac2) | **0.395** | 0.075 | **0.432** | 0.211 | **0.343** | 0.060 | **0.388** | 0.190 | 0.856 | 0.773 | 0.970 | 0.802 |
| NM_013022 | Rho-associated coiled-coil forming kinase 2 (Rock2) | 0.796 | 0.110 | **0.403** | **0.000** | **0.559** | **0.007** | **0.391** | **0.002** | 1.409 | **0.010** | 0.984 | 0.809 |
| NM_017322 | mitogen-activated protein kinase 9 (Mapk9) | **0.461** | **0.015** | **0.337** | **0.004** | **0.397** | **0.012** | **0.492** | **0.009** | 0.866 | 0.386 | 1.075 | 0.818 |
| NM_023962 | platelet-derived growth factor, D polypeptide (Pdgfd) | **0.137** | **0.000** | **0.320** | **0.010** | **0.137** | **0.000** | **0.274** | **0.008** | **0.429** | **0.000** | 0.857 | 0.825 |
| XM_342172 | thrombospondin 4 (Thbs4) | **0.126** | **0.005** | **0.092** | 0.090 | **0.155** | **0.006** | **0.101** | 0.092 | 1.412 | 0.592 | 0.917 | 0.861 |
| NM_030994 | integrin alpha 1 (Itga1) | **0.548** | 0.111 | **0.237** | 0.126 | **0.245** | **0.018** | **0.284** | 0.146 | 0.827 | 0.723 | 0.958 | 0.892 |
| NM_017022 | integrin beta 1 (fibronectin receptor beta) (Itgb1) | 0.925 | 0.847 | **0.455** | **0.012** | **0.528** | **0.000** | **0.411** | **0.002** | 1.315 | **0.036** | 1.023 | 0.902 |
| NM_031761 | c-fos induced growth factor (Figf) | **0.094** | **0.004** | **0.402** | **0.029** | **0.112** | **0.005** | **0.212** | **0.009** | **0.580** | **0.021** | 1.098 | 0.927 |
| **Regulation of actin cytoskeleton** | | n=9 | p<0.02 | n=16 | p<0.001 | n=20 | p<0.001 | n=23 | p<0.001 | n=3 |  | n=8 | p<0.001 |
| ACCESSION | Name | yMSCs | | aMSCs | | yMSCs | | aMSCs | | aMSCs/yMSCs | | aMSCs/yMSCs | |
|  |  | P30/P2 | | P30/P2 | | P100/P2 | | P100/P2 | | P2 | | P100 | |
|  |  | Ratio | p-value | Ratio | p-value | Ratio | p-value | Ratio | p-value | Ratio | p-value | Ratio | p-value |
| XM_213782 | actin related protein 2/3 complex, subunit 3 (Arpc3 ) | **0.448** | **0.002** | **0.555** | **0.005** | **0.431** | **0.000** | **0.389** | **0.002** | 1.017 | 0.896 | 0.919 | 0.657 |
| XM_238365 | actin related protein 2/3 complex, subunit 4 (Arpc4 ) | **0.610** | **0.002** | 0.890 | 0.398 | **0.549** | **0.000** | **0.612** | **0.016** | 1.073 | 0.585 | 1.196 | 0.207 |
| NM_019212 | actin, alpha 1, skeletal muscle (Acta1) | **0.091** | **0.024** | **0.645** | 0.128 | **0.023** | **0.019** | **0.004** | **0.001** | **2.167** | **0.002** | **0.382** | 0.075 |
| XM_345674 | cofilin 2, muscle (Cfl2 ) | 0.953 | 0.818 | **0.616** | **0.030** | **0.659** | **0.006** | **0.535** | **0.017** | 1.242 | 0.118 | 1.008 | 0.954 |
| NM_012951 | fibroblast growth factor 10 (Fgf10) | **0.249** | **0.007** | **0.177** | **0.004** | **0.249** | **0.010** | **0.152** | **0.004** | 1.404 | 0.198 | 0.857 | 0.794 |
| XM_341940 | fibroblast growth factor receptor 2 (Fgfr2) | **0.406** | **0.001** | **0.252** | **0.007** | **0.350** | **0.000** | **0.498** | **0.029** | **1.631** | **0.029** | **2.321** | **0.015** |
| NM_019143 | fibronectin 1 (Fn1) | 0.763 | 0.056 | **0.455** | **0.002** | **0.653** | **0.000** | **0.507** | **0.003** | 1.103 | 0.448 | 0.856 | 0.218 |
| XM_341851 | Harvey rat sarcoma oncogene, subgroup R (Rras ) | **0.534** | **0.004** | **0.635** | **0.042** | **0.386** | **0.000** | **0.388** | **0.004** | 1.163 | 0.245 | 1.171 | 0.438 |
| XM_579510 | hypothetical gene supported by NM_031525 (LOC497724) | 1.008 | 0.804 | 0.911 | 0.633 | **0.340** | **0.031** | **0.552** | **0.026** | 1.094 | 0.680 | **1.778** | **0.022** |
| XM_230033 | integrin alpha 4 (Itga4) | 1.259 | 0.229 | **1.792** | 0.100 | **0.126** | **0.000** | **0.195** | **0.011** | 0.840 | 0.439 | 1.302 | 0.701 |
| NM_017022 | integrin beta 1 (fibronectin receptor beta) (Itgb1) | 0.925 | 0.847 | **0.455** | **0.012** | **0.528** | **0.000** | **0.411** | **0.002** | 1.315 | **0.036** | 1.023 | 0.902 |
| XM_236320 | integrin, alpha 11 (Itga11 ) | 0.830 | 0.582 | 0.837 | 0.113 | **0.322** | **0.000** | **0.415** | **0.001** | 0.821 | 0.135 | 1.059 | 0.759 |
| XM_215984 | integrin, alpha 6 (Itga6) | **1.759** | 0.186 | **0.600** | 0.342 | 0.851 | 0.436 | **0.248** | **0.001** | **1.571** | 0.105 | **0.458** | 0.202 |
| XM_341877 | IQ motif containing GTPase activating protein 1 (Iqgap1 ) | 0.920 | 0.997 | **0.362** | **0.050** | **0.511** | 0.057 | **0.495** | **0.027** | 1.294 | 0.159 | 1.254 | 0.392 |
| NM_030863 | moesin (Msn) | **0.567** | **0.043** | **0.117** | **0.010** | **0.350** | **0.003** | **0.344** | **0.013** | **1.557** | **0.032** | **1.529** | **0.011** |
| XM_218617 | myosin, heavy polypeptide 14 (Myh14 ) | 1.000 | 1.000 | **1.740** | **0.032** | 1.000 | 1.000 | **0.251** | **0.012** | **2.140** | 0.089 | 0.890 | 0.561 |
| XM_215905 | myosin, light polypeptide 9, regulatory (Myl9 ) | **0.302** | **0.023** | **0.447** | **0.041** | **0.117** | **0.010** | **0.131** | **0.009** | 0.946 | 0.835 | 1.065 | 0.716 |
| XM_230038 | NCK-associated protein 1 (Nckap1) | **0.615** | 0.133 | **0.362** | **0.000** | **0.371** | **0.000** | **0.588** | **0.038** | 1.217 | 0.133 | **1.929** | **0.034** |
| NM_053306 | p21 (CDKN1A)-activated kinase 2 (Pak2) | **0.495** | 0.077 | **0.251** | **0.012** | **0.430** | **0.045** | **0.503** | **0.028** | 1.041 | 0.849 | 1.217 | 0.465 |
| NM_012801 | platelet derived growth factor, alpha (Pdgfa) | 0.684 | 0.058 | 0.874 | 0.333 | **0.620** | **0.008** | **0.562** | **0.008** | 0.811 | 0.112 | 0.734 | **0.016** |
| NM_057132 | ras homolog gene family, member A (Rhoa) | **0.161** | **0.001** | **0.573** | **0.040** | **0.260** | **0.001** | **0.250** | **0.001** | 1.162 | 0.248 | 1.117 | 0.743 |
| NM_031098 | Rho-associated coiled-coil forming kinase 1 (Rock1) | **0.504** | 0.133 | **0.162** | **0.003** | **0.366** | **0.050** | **0.395** | **0.013** | 1.305 | 0.205 | 1.407 | 0.476 |
| XM_344634 | similar to integrin alpha 8 (LOC364786) | **0.436** | 0.143 | **0.196** | **0.018** | **0.359** | **0.034** | **0.168** | **0.016** | **1.831** | 0.125 | 0.857 | 0.677 |
| NM_031727 | Rattus norvegicus LIM motif-containing protein kinase 1 (Limk1), mRNA. | 1.175 | **0.040** | **2.662** | 0.165 | 0.846 | 0.151 | **3.362** | **0.015** | 0.999 | 0.995 | **3.968** | **0.000** |
| NM_022511 | Rattus norvegicus profilin 1 (Pfn1), mRNA. | 0.892 | 0.525 | 0.838 | 0.312 | 0.828 | 0.219 | **0.651** | 0.062 | 0.981 | 0.886 | 0.771 | 0.153 |
| NM_030873 | Rattus norvegicus profilin 2 (Pfn2), mRNA. | **0.578** | **0.001** | **0.618** | **0.021** | **0.400** | **0.000** | **0.613** | **0.012** | 1.109 | 0.425 | **1.700** | **0.002** |
| NM_019131 | Rattus norvegicus tropomyosin 1, alpha (Tpm1), mRNA. | **1.675** | **0.032** | **1.734** | **0.019** | 1.222 | 0.175 | **0.597** | **0.050** | 0.934 | 0.737 | **0.456** | **0.000** |
| NM_057208 | Rattus norvegicus tropomyosin 3, gamma (Tpm3), transcript variant 1, mRNA. | **2.420** | **0.001** | **1.951** | **0.014** | **2.981** | **0.002** | 1.000 | 1.000 | 0.917 | 0.826 | **0.279** | **0.000** |
| NM_012678 | Rattus norvegicus tropomyosin 4 (Tpm4), mRNA. | 0.682 | 0.050 | **0.307** | **0.042** | **0.407** | **0.005** | **0.623** | **0.006** | 0.943 | 0.653 | 1.442 | **0.034** |
| **Mitochondrion (n=100, p<0.001)** | | n=15 | p<0.001 | n=5 | p<0.001 | n=22 | p<0.001 | n=11 | p<0.001 | n=5 |  | n=9 |  |
| ACCESSION | Name | yMSCs | | aMSCs | | yMSCs | | aMSCs | | aMSCs/yMSCs | | aMSCs/yMSCs | |
|  |  | P30/P2 | | P30/P2 | | P100/P2 | | P100/P2 | | P2 | | P100 | |
|  |  | Ratio | p-value | Ratio | p-value | Ratio | p-value | Ratio | p-value | Ratio | p-value | Ratio | p-value |
| NM_053995 | 3-hydroxybutyrate dehydrogenase (heart, mitochondrial) (Bdh), nuclear gene encoding mitochondrial protein | **0.511** | **0.017** | 0.907 | 0.422 | **0.328** | **0.005** | **0.544** | **0.005** | **0.610** | **0.006** | 1.011 | 0.953 |
| XM_573108 | A kinase (PRKA) anchor protein 10 (Akap10 ) | **0.350** | 0.071 | **0.368** | **0.016** | **0.301** | **0.012** | **0.453** | **0.001** | 0.805 | 0.556 | 1.209 | 0.783 |
| NM_012820 | acyl-CoA synthetase long-chain family member 1 (Acsl1) | 0.706 | 0.083 | 1.399 | 0.062 | **0.412** | **0.011** | **0.345** | **0.003** | 1.203 | 0.236 | 1.009 | 0.968 |
| NM_017135 | adenylate kinase 3-like 1 (Ak3l1) | **0.293** | **0.040** | **1.513** | 0.150 | **0.114** | **0.013** | **0.298** | **0.001** | 0.890 | 0.666 | **2.329** | **0.017** |
| NM_080890 | arsenic (+3 oxidation state) methyltransferase (As3mt) | **0.301** | **0.001** | 1.144 | 0.613 | **0.066** | **0.000** | **0.060** | **0.000** | 0.941 | 0.685 | 0.857 | 0.684 |
| NM_198745 | ATPase, H+ transporting, V1 subunit E isoform 1 (Atp6v1e1) | **0.506** | **0.001** | 0.749 | 0.085 | **0.375** | **0.000** | **0.379** | **0.001** | 0.951 | 0.703 | 0.962 | 0.767 |
| NM_013040 | ATP-binding cassette, sub-family C (CFTR/MRP), member 9 (Abcc9) | 0.736 | 0.468 | **0.146** | **0.000** | **0.453** | **0.024** | **0.332** | **0.000** | 1.014 | 0.942 | 0.742 | 0.314 |
| NM_021850 | Bcl2-like 2 (Bcl2l2) | 0.732 | 0.136 | 0.689 | 0.098 | **0.564** | **0.002** | **0.594** | **0.018** | 1.201 | 0.190 | 1.264 | 0.179 |
| NM_133561 | brain protein 44-like (Brp44l) | **0.393** | **0.015** | **0.577** | **0.033** | **0.366** | **0.011** | **0.407** | **0.008** | 0.908 | 0.562 | 1.009 | 0.971 |
| XM_214551 | cell death-inducing DNA fragmentation factor, alpha subunit-like effector A (Cidea ) | **0.409** | **0.000** | **0.292** | **0.020** | **0.187** | **0.000** | **0.125** | **0.009** | 1.031 | 0.898 | 0.691 | 0.574 |
| XM_217019 | ceramide kinase (Cerk ) | 0.691 | **0.027** | 0.837 | 0.242 | **0.523** | **0.001** | **0.498** | **0.003** | 1.196 | 0.178 | 1.139 | 0.383 |
| NM_199387 | CGI-12 protein (Cgi12) | **0.562** | **0.003** | **0.564** | **0.007** | **0.452** | **0.002** | **0.643** | **0.032** | 0.871 | 0.313 | 1.238 | 0.237 |
| NM_031818 | chloride intracellular channel 4 (Clic4) | **0.331** | **0.043** | **0.295** | **0.004** | **0.201** | **0.024** | **0.181** | **0.000** | 1.006 | 0.979 | 0.905 | 0.476 |
| XM_231402 | claudin 12 (Cldn12 ) | **0.354** | **0.000** | **0.619** | **0.001** | **0.021** | **0.000** | **0.515** | **0.000** | 0.990 | 0.938 | **24.216** | **0.000** |
| XM_238346 | coiled-coil-helix-coiled-coil-helix domain containing 3 (Chchd3 ) | 0.737 | **0.039** | 0.774 | **0.002** | **0.272** | **0.000** | **0.274** | **0.000** | 0.962 | 0.767 | 0.970 | 0.832 |
| NM_012529 | creatine kinase, brain (Ckb) | **0.399** | **0.001** | **0.158** | **0.006** | **0.094** | **0.000** | **0.003** | **0.003** | **1.837** | **0.005** | **0.059** | **0.000** |
| NM_024161 | cysteine string protein (Dnajc5) | **0.488** | **0.008** | 0.761 | **0.001** | **0.493** | **0.001** | **0.476** | **0.000** | 0.805 | 0.137 | 0.777 | 0.156 |
| NM_024160 | cytochrome b-245, alpha polypeptide (Cyba) | **0.458** | **0.005** | 1.004 | 0.934 | **0.388** | **0.000** | **0.007** | **0.000** | 0.841 | 0.188 | **0.014** | **0.000** |
| NM_017202 | cytochrome c oxidase subunit IV isoform 1 (Cox4i1) | **0.624** | **0.032** | 0.981 | 0.939 | **0.631** | **0.002** | **0.634** | **0.046** | 0.902 | 0.431 | 0.907 | 0.688 |
| NM_145783 | cytochrome c oxidase, subunit Va (Cox5a) | **0.573** | **0.022** | 0.813 | **0.042** | **0.635** | **0.003** | **0.541** | **0.044** | 0.788 | 0.072 | **0.671** | 0.150 |
| NM_012812 | cytochrome c oxidase, subunit VIa, polypeptide 2 (Cox6a2) | **0.283** | **0.002** | **0.430** | 0.083 | **0.118** | **0.000** | **0.165** | **0.033** | **0.356** | **0.000** | **0.498** | **0.029** |
| NM_012839 | cytochrome c, somatic (Cycs) | **0.663** | **0.017** | 0.714 | **0.008** | **0.583** | **0.003** | **0.569** | **0.010** | 0.801 | 0.094 | 0.782 | 0.129 |
| NM_012785 | demethyl-Q 7 (Coq7) | **0.374** | **0.007** | 0.860 | 0.231 | **0.487** | **0.017** | **0.387** | **0.009** | 0.829 | 0.594 | **0.659** | 0.536 |
| NM_053655 | dynamin 1-like (Dnm1l) | **0.661** | 0.149 | **0.431** | **0.003** | **0.490** | **0.004** | **0.534** | **0.041** | 1.116 | 0.424 | 1.218 | 0.510 |
| NM_001009668 | electron transferring flavoprotein, alpha polypeptide (Etfa) | **0.456** | **0.009** | **0.581** | **0.005** | **0.430** | **0.009** | **0.558** | **0.006** | 0.812 | 0.148 | 1.054 | 0.773 |
| XM_214838 | ethylmalonic encephalopathy 1 (Ethe1 ) | **0.495** | **0.004** | 0.895 | 0.456 | **0.388** | **0.001** | **0.370** | **0.001** | 1.146 | 0.303 | 1.092 | 0.626 |
| NM_024162 | fatty acid binding protein 3 (Fabp3) | **0.660** | 0.211 | 0.821 | **0.034** | **0.053** | **0.007** | **0.124** | **0.000** | 0.711 | 0.155 | **1.671** | 0.083 |
| NM_012569 | glutaminase (Gls) | **0.352** | **0.004** | **0.305** | **0.000** | **0.161** | **0.000** | **0.266** | **0.000** | 1.240 | 0.103 | **2.047** | **0.001** |
| NM_030826 | glutathione peroxidase 1 (Gpx1) | **0.485** | **0.004** | **0.385** | **0.002** | **0.520** | **0.000** | **0.513** | **0.011** | 0.834 | 0.056 | 0.823 | 0.250 |
| NM_181371 | glutathione S-transferase kappa 1 (Gstk1) | **0.275** | **0.010** | **0.158** | **0.000** | **0.018** | **0.003** | **0.148** | **0.000** | **0.622** | **0.032** | **5.180** | **0.000** |
| NM_001005908 | growth hormone inducible transmembrane protein (Ghitm) | **0.653** | **0.000** | 0.864 | 0.119 | **0.556** | **0.000** | **0.638** | **0.001** | 0.827 | 0.150 | 0.949 | 0.688 |
| NM_024487 | GrpE-like 1, mitochondrial (Grpel1) | **0.576** | **0.004** | 0.789 | **0.039** | **0.578** | **0.000** | **0.587** | **0.013** | 0.885 | 0.356 | 0.899 | 0.515 |
| NM_024377 | guanine nucleotide binding protein (G protein), gamma 5 subunit (Gng5) | **0.388** | **0.013** | **0.430** | **0.003** | **0.459** | **0.027** | **0.356** | **0.001** | 1.001 | 0.997 | 0.777 | 0.463 |
| XM_342966 | heat shock 27kD protein family, member 7 (cardiovascular) (Hspb7) | **0.059** | **0.049** | **0.064** | **0.016** | **0.059** | **0.048** | **0.028** | **0.014** | **1.772** | 0.167 | 0.857 | 0.962 |
| NM_021863 | heat shock protein 2 (Hspa2) | 0.901 | 0.403 | **0.618** | 0.052 | **0.574** | **0.001** | **0.555** | **0.004** | 1.355 | 0.057 | 1.311 | 0.248 |
| XM_342775 | heme binding protein 1 (Hebp1 ) | **0.483** | **0.003** | **0.574** | **0.005** | **0.521** | **0.001** | **0.648** | **0.049** | 0.871 | 0.296 | 1.085 | 0.693 |
| NM_033349 | hydroxyacyl glutathione hydrolase (Hagh) | **0.495** | 0.053 | **0.434** | **0.003** | **0.509** | **0.036** | **0.222** | **0.001** | **1.584** | **0.005** | 0.692 | **0.008** |
| NM_057186 | L-3-hydroxyacyl-Coenzyme A dehydrogenase, short chain (Hadhsc) | **0.488** | **0.001** | 0.924 | 0.484 | **0.372** | **0.000** | **0.415** | **0.000** | 0.806 | 0.115 | 0.901 | 0.487 |
| NM_013006 | lysophospholipase 1 (Lypla1) | **0.671** | **0.000** | **0.677** | **0.017** | **0.527** | **0.000** | **0.546** | **0.002** | 1.232 | 0.108 | 1.277 | 0.094 |
| XM_341880 | malic enzyme 3, NADP(+)-dependent, mitochondrial (Me3 ) | **0.296** | **0.002** | 0.729 | 0.553 | **0.296** | **0.001** | **0.360** | **0.000** | 0.785 | 0.419 | 0.954 | 0.372 |
| NM_001007637 | mitochondrial ribosomal protein L24 (mrpl24) | **0.599** | **0.022** | 0.702 | **0.034** | **0.481** | **0.000** | **0.664** | **0.005** | 0.799 | 0.111 | 1.103 | 0.559 |
| NM_053646 | N-acylsphingosine amidohydrolase 2 (Asah2) | **0.181** | **0.001** | **0.307** | **0.009** | **0.181** | **0.001** | **0.167** | **0.004** | 0.929 | 0.733 | 0.857 | 0.942 |
| XM_214570 | NADH dehydrogenase (ubiquinone) 1 alpha subcomplex, 2 (Ndufa2 ) | **0.453** | **0.002** | **0.555** | **0.002** | **0.461** | **0.000** | **0.450** | **0.045** | 0.852 | 0.222 | 0.832 | 0.637 |
| XM_341374 | Nedd4 family interacting protein 2 (Ndfip2 ) | **0.440** | **0.007** | **0.427** | **0.000** | **0.427** | **0.007** | **0.486** | **0.001** | 0.867 | 0.325 | 0.985 | 0.919 |
| NM_130756 | peroxisomal acyl-CoA thioesterase 1 (Pte1) | **0.569** | **0.040** | 0.853 | 0.080 | **0.554** | **0.020** | **0.510** | **0.000** | 1.016 | 0.918 | 0.934 | 0.659 |
| XM_345143 | procollagen, type IV, alpha 3 (Goodpasture antigen) binding protein (Col4a3bp ) | **0.551** | **0.041** | **0.371** | **0.004** | **0.443** | **0.006** | **0.368** | **0.000** | 1.155 | 0.298 | 0.958 | 0.831 |
| NM_031152 | RAB11a, member RAS oncogene family (Rab11a) | **0.533** | **0.000** | **0.531** | **0.001** | **0.451** | **0.000** | **0.495** | **0.000** | 1.124 | 0.373 | 1.236 | 0.188 |
| NM_080580 | RAB3D, member RAS oncogene family (Rab3d) | **0.317** | **0.001** | 0.877 | 0.440 | **0.308** | **0.001** | **0.515** | **0.003** | 1.261 | 0.079 | **2.108** | **0.000** |
| XM_224993 | similar to 1-acylglycerolphosphate acyltransferase-epsilon (LOC306582) | **0.349** | **0.000** | **0.308** | **0.015** | **0.265** | **0.000** | **0.314** | **0.002** | 0.889 | 0.388 | 1.053 | 0.845 |
| XM_215537 | similar to armadillo repeat-containing protein (LOC294948) | **0.633** | **0.032** | **0.599** | **0.008** | **0.510** | **0.005** | **0.583** | **0.012** | 1.032 | 0.812 | 1.180 | 0.391 |
| XM_215428 | similar to chromosome 10 open reading frame 42 (LOC294560) | 0.727 | 0.327 | **0.259** | **0.020** | **0.383** | **0.028** | **0.293** | **0.003** | 1.188 | 0.369 | 0.909 | 0.658 |
| XM_341573 | similar to FUN14 domain containing 2 (LOC361288) | **0.436** | **0.001** | **0.421** | **0.002** | **0.318** | **0.000** | **0.215** | **0.000** | 1.111 | 0.435 | 0.751 | 0.106 |
| XM_573196 | similar to hypothetical protein D11Ertd99e (LOC498000) | **0.640** | **0.028** | 0.857 | **0.048** | **0.532** | **0.000** | **0.507** | **0.018** | 0.971 | 0.820 | 0.925 | 0.749 |
| NM_001009705 | similar to hypothetical protein FLJ10241 (MGC109149) | **0.313** | **0.024** | **0.494** | **0.001** | **0.296** | **0.023** | **0.460** | **0.000** | 0.964 | 0.870 | 1.498 | **0.011** |
| XM_235581 | similar to leucine-rich repeat kinase 2 (LOC300160) | **0.651** | 0.234 | **0.552** | **0.008** | **0.237** | **0.000** | **0.393** | **0.000** | 1.490 | **0.005** | **2.464** | **0.001** |
| XM_576439 | similar to mitochondrial ribosomal protein L41 (LOC501028) | **0.507** | **0.005** | **0.679** | **0.010** | **0.491** | **0.000** | **0.590** | **0.045** | 0.831 | 0.162 | 0.999 | 0.996 |
| XM_343760 | similar to NADH dehydrogenase (LOC363441) | **0.447** | **0.003** | 0.746 | 0.094 | **0.405** | **0.000** | **0.436** | **0.048** | 0.867 | 0.275 | 0.932 | 0.873 |
| XM_213811 | similar to nitrogen fixation cluster-like (LOC288740) | **0.625** | **0.028** | **0.575** | **0.001** | **0.482** | **0.001** | **0.551** | **0.017** | 0.989 | 0.930 | 1.130 | 0.567 |
| XM_344957 | similar to RIKEN cDNA 4930404J24 (LOC365361) | **0.450** | **0.005** | **0.362** | **0.008** | **0.458** | **0.001** | **0.485** | **0.045** | 1.082 | 0.662 | 1.147 | 0.713 |
| XM_573117 | similar to SCO cytochrome oxidase deficient homolog 1 (yeast) (LOC497930) | **0.350** | **0.007** | **0.524** | **0.039** | **0.398** | **0.006** | **0.441** | **0.022** | 0.823 | 0.285 | 0.910 | 0.776 |
| XM_341209 | similar to type IV putative aminophospholipid transporting ATPase (LOC360932) | **0.245** | **0.002** | **0.292** | **0.005** | **0.245** | **0.000** | **0.250** | **0.005** | 0.838 | 0.491 | 0.857 | 0.782 |
| **Oxidative phosphorylation (n=22, p<0.001)** | | n=15 | p<0.001 | n=5 | p<0.001 | n=22 | p<0.001 | n=11 | p<0.001 | n=1 |  | n=2 |  |
| ACCESSION | Name | yMSCs | | aMSCs | | yMSCs | | aMSCs | | aMSCs/yMSCs | | aMSCs/yMSCs | |
|  |  | P30/P2 | | P30/P2 | | P100/P2 | | P100/P2 | | P2 | | P100 | |
|  |  | Ratio | p-value | Ratio | p-value | Ratio | p-value | Ratio | p-value | Ratio | p-value | Ratio | p-value |
| NM_053578 | ATPase, H+ transporting, V0 subunit E isoform 1 (Atp6v0e1) | **0.489** | **0.007** | **0.665** | **0.004** | **0.414** | **0.001** | **0.416** | **0.007** | 1.032 | 0.806 | 1.038 | 0.839 |
| XM_340987 | ATPase, H+ transporting, V1 subunit A, isoform 1 | **0.546** | 0.119 | **0.201** | **0.005** | **0.399** | **0.021** | **0.527** | **0.031** | 0.915 | 0.637 | 1.208 | 0.484 |
| XM_576255 | ATPase, H+ transporting, V1 subunit C, isoform 1 (Atp6v1c1 ) | **0.455** | **0.003** | **0.365** | **0.000** | **0.389** | **0.003** | **0.441** | **0.000** | 1.008 | 0.832 | 1.142 | 0.349 |
| NM_198745 | ATPase, H+ transporting, V1 subunit E isoform 1 (Atp6v1e1) | **0.506** | **0.001** | 0.749 | 0.085 | **0.375** | **0.000** | **0.379** | **0.001** | 0.951 | 0.703 | 0.962 | 0.767 |
| NM_053884 | ATPase, H+ transporting, V1 subunit F (Atp6v1f) | **0.493** | **0.011** | 0.694 | **0.020** | **0.457** | **0.000** | **0.431** | **0.019** | 0.832 | 0.163 | 0.785 | 0.437 |
| NM_017202 | cytochrome c oxidase subunit IV isoform 1 (Cox4i1) | **0.624** | **0.032** | 0.981 | 0.939 | **0.631** | **0.002** | **0.634** | **0.046** | 0.902 | 0.431 | 0.907 | 0.688 |
| NM_182819 | cytochrome c oxidase subunit VIIb (Cox7b) | **0.643** | **0.031** | 0.845 | 0.255 | **0.642** | **0.005** | **0.652** | 0.239 | 0.904 | 0.438 | 0.917 | 0.782 |
| NM_145783 | cytochrome c oxidase, subunit Va (Cox5a) | **0.573** | **0.022** | 0.813 | **0.042** | **0.635** | **0.003** | **0.541** | **0.044** | 0.788 | 0.072 | **0.671** | 0.150 |
| NM_012812 | cytochrome c oxidase, subunit VIa,(Cox6a2) | **0.283** | **0.002** | **0.430** | 0.083 | **0.118** | **0.000** | **0.165** | **0.033** | **0.356** | **0.000** | **0.498** | **0.029** |
| NM_053540 | cytochrome c oxidase, subunit XVII assembly protein homolog (yeast) (Cox17) | 0.732 | 0.233 | 1.059 | 0.572 | **0.600** | 0.065 | **0.263** | **0.004** | 1.095 | 0.599 | **0.480** | **0.010** |
| NM_199495 | NADH dehydrogenase (ubiquinone) 1 alpha subcomplex 10 (Ndufa10) | 0.718 | **0.001** | 1.014 | 0.732 | **0.647** | **0.000** | 0.808 | **0.006** | 0.993 | 0.960 | 1.241 | 0.135 |
| NM_012985 | NADH dehydrogenase (ubiquinone) 1 alpha subcomplex 5 (Ndufa5) | **0.639** | **0.029** | 0.903 | 0.482 | **0.525** | **0.000** | **0.488** | 0.069 | 0.936 | 0.614 | 0.871 | 0.718 |
| XM_214570 | NADH dehydrogenase (ubiquinone) 1 alpha subcomplex, 2 (predicted) | **0.453** | **0.002** | **0.555** | **0.002** | **0.461** | **0.000** | **0.450** | **0.045** | 0.852 | 0.222 | 0.832 | 0.637 |
| XM_235518 | NADH dehydrogenase (ubiquinone) 1 alpha subcomplex, 6 (B14) | 0.720 | 0.147 | 0.886 | 0.390 | **0.611** | **0.001** | **0.644** | 0.240 | 0.910 | 0.471 | 0.960 | 0.907 |
| XM_217400 | NADH dehydrogenase (ubiquinone) 1 beta subcomplex 3 (predicted) | 0.694 | 0.083 | 0.829 | 0.127 | **0.639** | **0.017** | **0.623** | 0.165 | 1.071 | 0.596 | 1.046 | 0.898 |
| XM_215544 | NADH dehydrogenase (ubiquinone) 1 beta subcomplex, 5 (Ndufb5 ) | **0.646** | **0.024** | 0.903 | 0.271 | **0.639** | **0.000** | **0.641** | 0.113 | 0.883 | 0.342 | 0.885 | 0.630 |
| XM_341664 | NADH dehydrogenase (ubiquinone) 1 beta subcomplex, 7 (predicted) | 0.718 | 0.095 | 1.198 | 0.464 | **0.616** | **0.001** | **0.566** | 0.069 | 0.896 | 0.402 | 0.824 | 0.486 |
| XM_216929 | NADH dehydrogenase (ubiquinone) 1 beta subcomplex, 9 | 0.691 | 0.071 | 1.071 | 0.390 | **0.592** | **0.001** | **0.638** | 0.119 | 0.862 | 0.257 | 0.930 | 0.777 |
| NM_001009290 | NADH dehydrogenase (ubiquinone) 1, subcomplex unknown, 2 (Ndufc2) | 0.711 | **0.049** | 1.033 | 0.488 | **0.593** | **0.000** | 0.723 | 0.254 | 0.862 | 0.257 | 1.050 | 0.860 |
| NM_182671 | NADH dehydrogenase 1 alpha subcomplex 10-like protein (LOC316632) | **0.656** | **0.001** | 1.044 | 0.443 | **0.572** | **0.000** | 0.771 | **0.002** | 0.994 | 0.966 | 1.341 | **0.006** |
| NM_212516 | similar to CG6105-PA (MGC72942) | **0.496** | **0.006** | **0.596** | **0.002** | **0.486** | **0.000** | **0.517** | 0.071 | 0.882 | 0.340 | 0.939 | 0.869 |
| XM_343760 | similar to NADH dehydrogenase (LOC363441) | **0.447** | **0.003** | 0.746 | 0.094 | **0.405** | **0.000** | **0.436** | **0.048** | 0.867 | 0.275 | 0.932 | 0.873 |
| NM_198788 | succinate dehydrogenase complex, subunit D, integral membrane protein (Sdhd) | **0.603** | **0.004** | 0.952 | 0.613 | **0.593** | **0.005** | 0.774 | **0.042** | 0.921 | 0.530 | 1.202 | 0.242 |
| NM_001006970 | ubiquinol cytochrome c reductase core protein 2 (Uqcrc2) | **0.556** | **0.000** | 0.691 | **0.012** | **0.520** | **0.000** | 0.795 | **0.030** | 0.918 | 0.515 | 1.405 | **0.025** |
| XM_343225 | ubiquinol-cytochrome c reductase binding protein (Uqcrb ) | 0.752 | 0.141 | 1.036 | 0.608 | **0.559** | **0.001** | 0.685 | 0.315 | 0.856 | 0.236 | 1.049 | 0.640 |
| **Glutathione metabolism (13, p<0.01)** | | n=11 | p<0.001 | n=9 | p<0.001 | n=13 | p<0.001 | n=8 | p<0.001 | n=5 | p<0.001 | n=7 | p<0.001 |
| ACCESSION | Name | yMSCs | | aMSCs | | yMSCs | | aMSCs | | aMSCs/yMSCs | | aMSCs/yMSCs | |
|  |  | P30/P2 | | P30/P2 | | P100/P2 | | P100/P2 | | P2 | | P100 | |
|  |  | Ratio | p-value | Ratio | p-value | Ratio | p-value | Ratio | p-value | Ratio | p-value | Ratio | p-value |
| NM_181371 | Rattus norvegicus glutathione S-transferase kappa 1 (Gstk1), mRNA. | **0.275** | **0.010** | **0.158** | **0.000** | **0.018** | **0.003** | **0.148** | **0.000** | **0.622** | **0.032** | **5.180** | **0.000** |
| NM_177426 | Rattus norvegicus glutathione S-transferase, mu 2 (Gstm2), mRNA. | **0.251** | **0.000** | **0.169** | **0.000** | **0.298** | **0.000** | **0.509** | **0.013** | 0.889 | 0.374 | **1.520** | 0.063 |
| XM_215562 | PREDICTED: Rattus norvegicus microsomal glutathione S-transferase 2 (Mgst2 ), mRNA. | **0.362** | **0.011** | **1.956** | **0.009** | **0.544** | **0.028** | **3.439** | **0.050** | **0.294** | **0.000** | **1.858** | 0.115 |
| NM_134349 | Rattus norvegicus microsomal glutathione S-transferase 1 (Mgst1), mRNA. | **0.147** | **0.026** | **0.075** | **0.007** | **0.038** | **0.018** | **8.665** | **0.014** | **0.244** | **0.011** | **55.446** | **0.000** |
| NM_031012 | Rattus norvegicus alanyl (membrane) aminopeptidase (Anpep), mRNA. | **0.042** | **0.005** | **0.237** | **0.011** | **0.019** | **0.004** | **0.546** | 0.106 | **0.532** | **0.016** | **15.182** | **0.002** |
| NM_053293 | Rattus norvegicus glutathione S-transferase theta 1 (Gstt1), mRNA. | **0.461** | **0.003** | 1.483 | 0.199 | **0.443** | **0.000** | **1.505** | 0.205 | 0.775 | 0.105 | **2.637** | **0.002** |
| XM_216482 | PREDICTED: Rattus norvegicus similar to RIKEN cDNA 0610040B21 | 0.758 | 0.074 | 0.713 | **0.000** | **0.660** | **0.008** | **0.613** | **0.003** | 0.999 | 0.994 | 0.928 | 0.564 |
| NM_030826 | Rattus norvegicus glutathione peroxidase 1 (Gpx1), mRNA. | **0.485** | **0.004** | **0.385** | **0.002** | **0.520** | **0.000** | **0.513** | **0.011** | 0.834 | 0.056 | 0.823 | 0.250 |
| NM_022525 | Rattus norvegicus glutathione peroxidase 3 (Gpx3), mRNA. | **0.011** | **0.001** | **0.228** | **0.020** | **0.005** | **0.001** | **0.004** | **0.008** | **1.619** | 0.081 | 1.499 | 0.299 |
| NM_172038 | Rattus norvegicus glutathione S-transferase, mu 5 (Gstm5), mRNA. | **0.289** | **0.001** | **0.660** | **0.035** | **0.255** | **0.001** | **0.333** | **0.003** | 0.683 | **0.006** | 0.893 | 0.573 |
| NM_012796 | Rattus norvegicus glutathione S-transferase, theta 2 (Gstt2), mRNA. | **0.394** | **0.002** | 0.932 | 0.418 | **0.238** | **0.000** | 0.854 | 0.133 | 0.866 | 0.295 | **3.113** | **0.000** |
| NM_031510 | Rattus norvegicus isocitrate dehydrogenase 1 (NADP+), soluble (Idh1), mRNA. | **0.508** | 0.051 | **0.387** | **0.006** | **0.372** | **0.018** | 0.918 | 0.736 | 1.178 | 0.373 | **2.904** | **0.000** |
| XM_579385 | PREDICTED: Rattus norvegicus glucose-6-phosphate dehydrogenase (G6pdx), mRNA. | **0.628** | **0.022** | 1.218 | 0.584 | **0.350** | **0.000** | 1.143 | 0.590 | **0.530** | **0.001** | **1.731** | **0.025** |
| **Wnt signaling pathway n=31, p<0.001)** | | n=17 | p<0.001 | n=17 | p<0.001 | n=31 | p<0.001 | n=23 | p<0.001 | n=6 | p<0.001 | n=11 | p<0.001 |
| ACCESSION | Name | yMSCs | | aMSCs | | yMSCs | | aMSCs | | aMSCs/yMSCs | | aMSCs/yMSCs | |
|  |  | P30/P2 | | P30/P2 | | P100/P2 | | P100/P2 | | P2 | | P100 | |
|  |  | Ratio | p-value | Ratio | p-value | Ratio | p-value | Ratio | p-value | Ratio | p-value | Ratio | p-value |
| NM_053615 | casein kinase 1, alpha 1 (Csnk1a1) | **0.620** | **0.017** | **0.526** | **0.018** | **0.575** | **0.004** | 0.810 | 0.356 | 1.036 | 0.868 | 1.459 | 0.285 |
| XM_220281 | F-box and WD-40 domain protein 11 (Fbxw11 ) | **0.329** | **0.025** | **0.234** | **0.006** | **0.354** | **0.005** | **0.514** | **0.016** | 0.990 | 0.965 | 1.440 | 0.511 |
| NM_021266 | frizzled homolog 1 (Drosophila) (Fzd1) | **0.244** | **0.000** | **0.130** | **0.001** | **0.072** | **0.000** | **0.299** | **0.002** | 1.177 | 0.239 | **4.896** | **0.000** |
| XM_237191 | frizzled homolog 7 (Drosophila) (Fzd7 ) | **0.429** | **0.003** | **0.136** | **0.001** | **0.300** | **0.000** | **0.232** | **0.000** | **2.055** | **0.000** | **1.589** | 0.248 |
| XM_215187 | low density lipoprotein receptor-related protein 5 (Lrp5 ) | **0.628** | 0.163 | **0.599** | **0.009** | 0.811 | **0.044** | **0.275** | **0.001** | 1.333 | 0.066 | **0.452** | **0.001** |
| NM_019191 | MAD homolog 2 (Drosophila) (Smad2) | 0.709 | **0.035** | 0.883 | 0.460 | **0.634** | **0.015** | 0.826 | 0.120 | 1.031 | 0.817 | 1.345 | 0.061 |
| NM_017322 | mitogen-activated protein kinase 9 (Mapk9) | **0.461** | **0.015** | **0.337** | **0.004** | **0.397** | **0.012** | **0.492** | **0.009** | 0.866 | 0.386 | 1.075 | 0.818 |
| XM_344728 | naked cuticle 1 homolog (Drosophila) (Nkd1 ) | **0.179** | **0.011** | **n.d** | n.d | **0.179** | **0.011** | **n.d** | n.d | **0.275** | **0.003** | 1.336 | 0.593 |
| XM_240184 | nuclear factor of activated T-cells, cytoplasmic, calcineurin-dependent 4 (Nfatc4 ) | 0.943 | 0.668 | 0.931 | 0.768 | **0.304** | **0.008** | 1.429 | 0.050 | 1.026 | 0.885 | **4.825** | **0.000** |
| XM_235609 | prickle-like 1 (Drosophila) (Prickle1) | 1.136 | 0.374 | **0.554** | 0.059 | **0.364** | **0.000** | 1.120 | 0.374 | 0.999 | 0.994 | **3.073** | **0.000** |
| XM_343975 | protein kinase C, alpha (Prkca) | 0.935 | 0.703 | 0.729 | **0.021** | 0.819 | 0.220 | **0.546** | **0.011** | 1.384 | **0.041** | 0.923 | 0.716 |
| NM_012713 | protein kinase C, beta 1 (Prkcb1) | **0.086** | **0.039** | **0.135** | 0.174 | **0.086** | **0.044** | **0.115** | 0.176 | **0.637** | 0.562 | 0.857 | 0.866 |
| NM_181379 | protein phosphatase 2, regulatory subunit B (B56), beta isoform (Ppp2r5b) | **0.469** | **0.024** | **0.257** | 0.059 | **0.403** | **0.006** | **0.591** | **0.010** | 0.736 | 0.052 | 1.079 | 0.707 |
| NM_017042 | protein phosphatase 3, catalytic subunit, beta isoform (Ppp3cb) | 0.959 | 0.975 | **0.282** | **0.017** | **0.475** | **0.006** | 0.748 | 0.218 | 1.156 | 0.288 | **1.821** | **0.041** |
| NM_017309 | protein phosphatase 3, regulatory subunit B, alpha isoform (calcineurin B, type I) (Ppp3r1) | 0.682 | **0.006** | 0.782 | 0.079 | **0.586** | **0.000** | **0.586** | **0.003** | 1.027 | 0.836 | 1.026 | 0.850 |
| NM_057132 | ras homolog gene family, member A (Rhoa) | **0.161** | **0.001** | **0.573** | **0.040** | **0.260** | **0.001** | **0.250** | **0.001** | 1.162 | 0.248 | 1.117 | 0.743 |
| NM_031098 | Rho-associated coiled-coil forming kinase 1 (Rock1) | **0.504** | 0.133 | **0.162** | **0.003** | **0.366** | **0.050** | **0.395** | **0.013** | 1.305 | 0.205 | 1.407 | 0.476 |
| NM_013022 | Rho-associated coiled-coil forming kinase 2 (Rock2) | 0.796 | 0.110 | **0.403** | **0.000** | **0.559** | **0.007** | **0.391** | **0.002** | 1.409 | **0.010** | 0.984 | 0.939 |
| NM_053544 | secreted frizzled-related protein 4 (Sfrp4) | **0.036** | **0.003** | **0.089** | **0.007** | **0.036** | **0.003** | **0.492** | 0.082 | **0.404** | **0.002** | **5.528** | **0.003** |
| NM_080905 | seven in absentia 1A (Siah1a) | 0.910 | 0.604 | 0.755 | **0.044** | **0.620** | **0.039** | 0.942 | 0.572 | 0.966 | 0.818 | 1.468 | **0.023** |
| XM_575321 | similar to calcium binding protein P22 (LOC499969) | **0.497** | **0.010** | **0.548** | **0.013** | **0.371** | **0.000** | **0.551** | **0.048** | 0.919 | 0.527 | 1.364 | 0.326 |
| XM_575825 | similar to calcium binding protein P22 (LOC500462) | **0.613** | **0.024** | **0.649** | **0.027** | **0.495** | **0.000** | 0.777 | 0.264 | 0.887 | 0.363 | 1.393 | 0.177 |
| NM_001004208 | similar to calcyclin binding protein (MGC93921) | **0.658** | **0.000** | 1.014 | 0.982 | **0.658** | **0.001** | 0.998 | 0.949 | 0.890 | 0.374 | 1.350 | **0.048** |
| XM_345195 | similar to IRA1 protein (LOC365755) | **0.376** | **0.018** | **0.376** | **0.004** | **0.352** | **0.010** | 0.681 | 0.099 | 1.077 | 0.672 | **2.083** | **0.017** |
| XM_228781 | similar to porcupine-D (LOC317368) | **0.211** | **0.001** | **0.417** | **0.046** | **0.153** | **0.001** | **0.545** | 0.098 | **0.472** | **0.000** | **1.677** | **0.005** |
| XM_215070 | similar to protein kinase, cAMP dependent, catalytic, beta (LOC293508) | 0.845 | 0.123 | 0.947 | 0.690 | **0.555** | **0.001** | 0.977 | 0.730 | 1.018 | 0.892 | **1.794** | **0.001** |
| XM_344616 | similar to transmembrane receptor (LOC364754) | **0.574** | **0.018** | **0.428** | **0.011** | **0.183** | **0.001** | **0.322** | **0.005** | **1.592** | **0.006** | **2.806** | **0.000** |
| XM_575403 | similar to wingless-related MMTV integration site 16 (LOC500047) | **0.126** | **0.018** | **n.d** | n.d | **0.126** | **0.015** | **n.d** | n.d | **0.090** | **0.000** | 0.857 | 0.973 |
| NM_001007608 | S-phase kinase-associated protein 1A (Skp1a) | 0.738 | **0.047** | 0.750 | **0.019** | **0.660** | **0.003** | 0.719 | **0.048** | 1.034 | 0.799 | 1.125 | 0.400 |
| XM_343891 | transcription factor 7, T-cell specific (Tcf7 ) | **1.511** | 0.193 | 1.281 | 0.335 | **0.287** | **0.008** | **2.176** | **0.026** | 1.045 | 0.856 | **7.911** | **0.000** |
| NM_022631 | wingless-type MMTV integration site 5A (Wnt5a) | **0.577** | 0.189 | **0.429** | **0.014** | **0.175** | **0.043** | **0.404** | **0.010** | **0.613** | 0.216 | 1.421 | 0.295 |
| **TGF/BMP-singaling (n=31, p<0.001)** | | n=17 | p<0.001 | n=17 | p<0.001 | n=31 | p<0.001 | n=23 | p<0.001 | n=7 | p<0.001 | n=17 | p<0.001 |
| ACCESSION | Name | yMSCs | | aMSCs | | yMSCs | | aMSCs | | aMSCs/yMSCs | | aMSCs/yMSCs | |
|  |  | P30/P2 | | P30/P2 | | P100/P2 | | P100/P2 | | P2 | | P100 | |
|  |  | Ratio | p-value | Ratio | p-value | Ratio | p-value | Ratio | p-value | Ratio | p-value | Ratio | p-value |
| NM_022441 | activin A receptor type II-like 1 (Acvrl1) | **0.036** | **0.002** | **0.058** | **0.001** | **0.016** | **0.002** | **0.014** | **0.001** | 0.953 | 0.789 | 0.826 | 0.672 |
| NM_024486 | activin A receptor, type 1 (Acvr1) | 0.715 | 0.227 | **0.264** | **0.036** | **0.370** | **0.002** | **0.636** | 0.187 | 0.981 | 0.931 | **1.686** | **0.022** |
| NM_139082 | BMP and activin membrane-bound inhibitor, homolog (Xenopus laevis) (Bambi) | **0.587** | 0.098 | 0.743 | 0.171 | **0.310** | **0.022** | **0.461** | **0.008** | **0.613** | 0.060 | 0.911 | 0.495 |
| NM_173115 | BMP/retinoic acid-inducible neural-specific protein 2 (Brinp2) | **0.369** | **0.020** | **0.295** | **0.003** | **0.369** | **0.024** | **0.253** | **0.002** | 1.250 | 0.489 | 0.857 | 0.802 |
| NM_017178 | bone morphogenetic protein 2 (Bmp2) | **0.273** | **0.009** | 0.836 | 0.531 | **0.189** | **0.003** | **2.851** | **0.002** | 0.954 | 0.825 | **14.371** | **0.000** |
| NM_017105 | bone morphogenetic protein 3 (Bmp3) | 0.759 | 0.126 | 0.873 | 0.422 | **0.521** | **0.031** | **0.672** | **0.038** | **0.680** | **0.042** | 0.877 | 0.565 |
| XM_236415 | bone morphogenetic protein 5 (Bmp5 ) | **0.290** | **0.022** | 1.000 | 1.000 | **0.290** | **0.019** | 1.000 | 1.000 | **0.207** | **0.011** | 0.857 | 0.827 |
| NM_013107 | bone morphogenetic protein 6 (Bmp6) | **0.318** | **0.002** | **0.145** | **0.000** | **0.245** | **0.001** | **0.118** | **0.000** | **1.785** | **0.005** | 0.857 | 0.994 |
| NM_030849 | bone morphogenetic protein receptor, type 1A (Bmpr1a) | **0.668** | 0.070 | **0.433** | **0.002** | **0.529** | **0.001** | 0.805 | **0.027** | 1.082 | 0.567 | **1.646** | **0.005** |
| XM_221307 | chordin (Chrd) | **0.410** | **0.037** | **0.665** | 0.155 | **0.314** | **0.019** | **1.684** | **0.045** | **0.663** | 0.165 | **3.550** | **0.001** |
| NM_130812 | cyclin-dependent kinase inhibitor 2B (p15, inhibits CDK4) (Cdkn2b) | **0.193** | **0.006** | **0.178** | **0.003** | **0.193** | **0.005** | **0.253** | **0.005** | 1.086 | 0.731 | 1.426 | 0.223 |
| NM_024129 | decorin (Dcn) | **0.007** | **0.030** | **0.061** | **0.017** | **0.007** | **0.030** | **0.240** | **0.038** | **0.112** | **0.003** | **3.906** | **0.000** |
| NM_012561 | follistatin (Fst) | **0.134** | **0.010** | 1.230 | 0.602 | **0.492** | **0.009** | 1.213 | 0.637 | 0.878 | 0.726 | **2.167** | **0.000** |
| NM_017128 | inhibin beta-A (Inhba) | **0.663** | 0.116 | **0.651** | 0.249 | **0.086** | **0.002** | **0.201** | **0.000** | 1.316 | 0.090 | **3.081** | **0.008** |
| NM_013060 | Inhibitor of DNA binding 2, dominant negative helix-loop-helix protein (Id2) | **0.252** | **0.049** | **0.371** | **0.003** | **0.077** | **0.027** | **0.263** | **0.001** | **1.782** | **0.013** | **6.087** | **0.000** |
| NM_021587 | latent transforming growth factor beta binding protein 1 (Ltbp1) | 0.886 | 0.748 | **0.626** | **0.041** | **0.605** | **0.005** | 1.173 | 0.345 | 0.981 | 0.896 | **1.903** | **0.000** |
| NM_013130 | MAD homolog 1 (Drosophila) (Smad1) | 0.875 | 0.088 | 0.698 | 0.195 | **0.493** | **0.000** | **0.631** | **0.048** | 1.232 | 0.323 | **1.579** | **0.006** |
| NM_019191 | MAD homolog 2 (Drosophila) (Smad2) | 0.709 | **0.035** | 0.883 | 0.460 | **0.634** | **0.015** | 0.826 | 0.120 | 1.031 | 0.817 | 1.345 | 0.061 |
| NM_053842 | mitogen activated protein kinase 1 (Mapk1) | 0.710 | **0.006** | **0.624** | **0.001** | **0.628** | **0.002** | **0.558** | **0.001** | 1.166 | 0.241 | 1.036 | 0.799 |
| NM_017347 | mitogen activated protein kinase 3 (Mapk3) | **0.651** | **0.007** | 0.802 | 0.646 | **0.528** | **0.002** | 1.176 | 0.110 | 1.105 | 0.446 | **2.461** | **0.000** |
| XM_343954 | PREDICTED: Rattus norvegicus noggin (Nog), mRNA. | **0.185** | 0.170 | **0.130** | **0.016** | **0.193** | 0.179 | **0.159** | **0.020** | 0.947 | 0.935 | 0.780 | 0.725 |
| NM_057132 | ras homolog gene family, member A (Rhoa) | **0.161** | **0.001** | **0.573** | **0.040** | **0.260** | **0.001** | **0.250** | **0.001** | 1.162 | 0.248 | 1.117 | 0.743 |
| NM_031094 | retinoblastoma-like 2 (Rbl2) | **0.521** | **0.003** | 0.729 | 0.089 | **0.327** | **0.000** | 0.929 | 0.843 | 0.856 | 0.300 | **2.434** | **0.000** |
| NM_031098 | Rho-associated coiled-coil forming kinase 1 (Rock1) | **0.504** | 0.133 | **0.162** | **0.003** | **0.366** | **0.050** | **0.395** | **0.013** | 1.305 | 0.205 | 1.407 | 0.476 |
| NM_013022 | Rho-associated coiled-coil forming kinase 2 (Rock2) | 0.796 | 0.110 | **0.403** | **0.000** | **0.559** | **0.007** | **0.391** | **0.002** | 1.409 | **0.010** | 0.984 | 0.939 |
| NM_031985 | ribosomal protein S6 kinase, polypeptide 1 (Rps6kb1) | **0.466** | **0.003** | **0.648** | **0.041** | **0.394** | **0.002** | **0.611** | **0.029** | 0.876 | 0.330 | 1.360 | 0.082 |
| XM_214778 | thrombospondin 2 (Thbs2 ) | **0.112** | **0.001** | **0.021** | **0.004** | **0.004** | **0.001** | **0.399** | **0.029** | **0.516** | **0.001** | **50.789** | **0.000** |
| XM_342172 | thrombospondin 4 (Thbs4) | **0.126** | **0.005** | **0.092** | 0.090 | **0.155** | **0.006** | **0.101** | 0.092 | 1.412 | 0.592 | 0.917 | 0.861 |
| NM_012671 | transforming growth factor alpha (Tgfa) | 1.071 | 0.579 | **0.567** | **0.008** | 0.691 | **0.029** | **0.171** | **0.000** | 1.055 | 0.679 | **0.260** | **0.000** |
| XM_341934 | transforming growth factor beta 1 induced transcript 1 (Tgfb1i1) | 1.000 | 1.000 | 1.069 | 0.898 | 1.000 | 1.000 | **0.487** | **0.026** | 1.359 | 0.382 | 1.073 | 0.923 |
| NM_021578 | transforming growth factor, beta 1 (Tgfb1) | 0.927 | 0.679 | 1.326 | 0.278 | **0.609** | **0.048** | 1.184 | 0.608 | 0.685 | 0.156 | 1.331 | 0.062 |
| NM_031131 | transforming growth factor, beta 2 (Tgfb2) | 1.291 | 0.095 | 1.020 | 0.919 | **0.433** | **0.001** | **0.244** | **0.009** | 1.194 | 0.358 | **0.674** | **0.002** |
| NM_013174 | transforming growth factor, beta 3 (Tgfb3) | 1.214 | 0.228 | **0.486** | **0.036** | 0.856 | **0.042** | **0.197** | **0.007** | **1.643** | **0.017** | **0.378** | **0.000** |
| XM_573983 | transforming growth factor, beta induced, 68 kDa (Tgfbi) | **5.988** | **0.015** | 1.000 | 1.000 | **18.972** | **0.000** | **29.188** | **0.007** | **0.475** | 0.134 | 0.731 | 0.109 |
| XM_237113 | transforming growth factor, beta receptor associated protein 1 (Tgfbrap1 ) | 1.000 | 1.000 | 1.000 | 1.000 | **1.680** | **0.000** | 1.000 | 1.000 | 0.735 | 0.485 | 0.699 | 0.310 |
| XM_217409 | bone morphogenic protein receptor, type II (Bmpr2) | 1.484 | 0.103 | 0.770 | **0.008** | 0.788 | **0.032** | **0.594** | **0.009** | 1.263 | 0.085 | 0.979 | 0.608 |
| **PPAR signaling pathway (n=9, p=0.04)** | | n=5 | p<0.001 | n=5 | p<0.001 | n=9 | p<0.001 | n=9 | p<0.001 | n=2 |  | n=3 |  |
| ACCESSION | Name | yMSCs | | aMSCs | | yMSCs | | aMSCs | | aMSCs/yMSCs | | aMSCs/yMSCs | |
|  |  | P30/P2 | | P30/P2 | | P100/P2 | | P100/P2 | | P2 | | P100 | |
|  |  | Ratio | p-value | Ratio | p-value | Ratio | p-value | Ratio | p-value | Ratio | p-value | Ratio | p-value |
| NM_012820 | Rattus norvegicus acyl-CoA synthetase long-chain family member 1 (Acsl1), mRNA. | 0.706 | 0.083 | 1.399 | 0.062 | **0.412** | **0.011** | **0.345** | **0.003** | 1.203 | 0.236 | 1.009 | 0.968 |
| NM_024162 | Rattus norvegicus fatty acid binding protein 3 (Fabp3), mRNA. | **0.660** | 0.211 | 0.821 | **0.034** | **0.053** | **0.007** | **0.124** | **0.000** | 0.711 | 0.155 | **1.671** | 0.083 |
| NM_012598 | Rattus norvegicus lipoprotein lipase (Lpl), mRNA. | **0.085** | **0.048** | **0.020** | **0.000** | **0.002** | **0.038** | **0.058** | **0.000** | **0.253** | **0.045** | **7.189** | **0.000** |
| NM_031627 | Rattus norvegicus nuclear receptor subfamily 1, group H, member 3 (Nr1h3), mRNA. | **0.074** | **0.011** | **0.179** | **0.012** | **0.074** | **0.010** | **0.078** | **0.007** | 0.817 | 0.569 | 0.857 | 0.582 |
| NM_133306 | Rattus norvegicus oxidized low density lipoprotein (lectin-like) receptor 1 (Oldlr1), mRNA. | **0.116** | **0.011** | **0.039** | **0.020** | **0.116** | **0.011** | **0.043** | **0.023** | **2.989** | **0.022** | 1.115 | 0.508 |
| NM_013124 | Rattus norvegicus peroxisome proliferator activated receptor gamma (Pparg), mRNA. | **0.177** | 0.061 | **0.175** | **0.000** | **0.040** | **0.037** | **0.192** | **0.000** | **0.456** | 0.115 | **2.205** | **0.018** |
| XM_215939 | PREDICTED: Rattus norvegicus phospholipid transfer protein (Pltp ), mRNA. | **0.118** | **0.002** | **1.726** | 0.132 | **0.071** | **0.002** | **0.301** | **0.002** | 0.707 | **0.037** | **2.999** | **0.000** |
| NM_053580 | Rattus norvegicus solute carrier family 27 (fatty acid transporter), member 1 (Slc27a1), mRNA. | **0.411** | **0.007** | 0.860 | 0.374 | **0.284** | **0.002** | **0.536** | **0.034** | 0.916 | 0.566 | **1.730** | 0.090 |
